# Retrospective evaluation of real-time estimates of global COVID-19 transmission trends and mortality forecasts

**DOI:** 10.1371/journal.pone.0286199

**Published:** 2023-10-18

**Authors:** Sangeeta Bhatia, Kris V. Parag, Jack Wardle, Rebecca K. Nash, Natsuko Imai, Sabine L. Van Elsland, Britta Lassmann, John S. Brownstein, Angel Desai, Mark Herringer, Kara Sewalk, Sarah Claire Loeb, John Ramatowski, Gina Cuomo-Dannenburg, Elita Jauneikaite, H. Juliette T. Unwin, Steven Riley, Neil Ferguson, Christl A. Donnelly, Anne Cori, Pierre Nouvellet

**Affiliations:** 1 MRC Centre for Global Infectious Disease Analysis, School of Public Health, Imperial College London, London, United Kingdom; 2 NIHR Health Protection Research Unit in Modelling and Health Economics, Modelling & Economics Unit, UK Health Security Agency, London, United Kingdom; 3 ProMED-mail, International Society for Infectious Diseases, Brookline, MA, United States of America; 4 Boston Children’s Hospital, Computational Epidemiology Lab, Boston, MA, United States of America; 5 Division of Infectious Diseases, Department of Internal Medicine, University of California Davis, Sacramento, California, United States of America; 6 Healthsites.io, The Global Healthsites Mapping Project, London, United Kingdom; 7 Department of Statistics, University of Oxford, Oxford, United Kingdom; 8 School of Life Sciences, University of Sussex, Brighton, United Kingdom; The Chinese University of Hong Kong, HONG KONG

## Abstract

Since 8^th^ March 2020 up to the time of writing, we have been producing near real-time weekly estimates of SARS-CoV-2 transmissibility and forecasts of deaths due to COVID-19 for all countries with evidence of sustained transmission, shared online. We also developed a novel heuristic to combine weekly estimates of transmissibility to produce forecasts over a 4-week horizon. Here we present a retrospective evaluation of the forecasts produced between 8^th^ March to 29^th^ November 2020 for 81 countries. We evaluated the robustness of the forecasts produced in real-time using relative error, coverage probability, and comparisons with null models. During the 39-week period covered by this study, both the short- and medium-term forecasts captured well the epidemic trajectory across different waves of COVID-19 infections with small relative errors over the forecast horizon. The model was well calibrated with 56.3% and 45.6% of the observations lying in the 50% Credible Interval in 1-week and 4-week ahead forecasts respectively. The retrospective evaluation of our models shows that simple transmission models calibrated using routine disease surveillance data can reliably capture the epidemic trajectory in multiple countries. The medium-term forecasts can be used in conjunction with the short-term forecasts of COVID-19 mortality as a useful planning tool as countries continue to relax public health measures.

## Introduction

As of June 2022, more than 6 million deaths have been attributed to COVID-19 with over 52 million cases reported globally [1]. The scale of the current pandemic has led to a widespread adoption of data-driven public health responses across the globe. Outbreak analysis and real-time modelling, including short-term forecasts of future incidence, have been used to inform decision making and response efforts in several past public health challenges including the West African Ebola epidemic and seasonal influenza [2–11]. In the current pandemic, mathematical models have helped public health officials better understand the evolving epidemiology of SARS-CoV-2 [12–14] and the potential impact of implementing or releasing interventions. Short-term forecasts of key indicators such as mortality, hospitalisation, and hospital occupancy have played a similarly important role [15–21], contributing to planning public health interventions and allocation of crucial resources [22–26]. At the same time, the unprecedented level of public interest has placed epidemiological modelling under intense media scrutiny. In light of the prominent role mathematical models have had in policy planning during the COVID-19 pandemic, retrospective assessment of modelling outputs against later empirical data is critical to assess their validity.

With the aim of improving situational awareness and providing a global overview during the ongoing pandemic, since the 8^th^ March 2020, we have been reporting weekly estimates of transmissibility of SARS-CoV-2 and forecasts of the daily incidence of deaths associated with COVID-19 for countries with evidence of sustained transmission [27] (published online https://mrc-ide.github.io/covid19-short-term-forecasts, and on the updated interface https://mriids.org). We have developed three models that were calibrated using the latest reported daily incidence of COVID-19 cases and deaths in each country. Transmissibility estimates and forecasts were based on an ensemble model comprising of the three models. Ensemble models, which combine outputs from different models, are a powerful way of incorporating the uncertainty from a range of models and can produce more robust forecasts than individual models [28–32].

Forecasts are typically produced under the assumption that the trend in growth remains the same over the forecast horizon. While this is a plausible assumption for the 1-week forecast horizon that we used for our short-term forecasts, it is likely to be violated over a longer forecast horizon. We have developed a novel approach relying on a simple heuristic that combines past estimates of the reproduction number, explicitly accounting for the predicted future changes in population immunity, to produce forecasts over longer time horizons.

Here we summarise the key transmission trends from our work on global short-term forecasts between 8^th^ March to 29^th^ November 2020. We provide a rigorous quantitative assessment of the performance of the ensemble model that was used to produce near real-time transmissibility estimates. We also present medium-term forecasts using our approach and retrospectively assess the performance of our method. Forecasts from infectious disease models of indicators such as incidence of cases or hospitalisations are used to inform a range of public health objectives. These objectives in turn determine model complexity, the data sources used for model calibration, and acceptable model performance thresholds. Since our work was aimed at generating situational awareness and provide a global overview of the pandemic, we implemented simple models calibrated using easily available data. As such, we did not specify *a priori* performance thresholds. Instead, we have used multiple metrics to explore model performance in relation to the underlying epidemiological situation.

## Methods

The instantaneous reproduction number is frequently used to quantify transmissibility. It is defined as the average number of secondary cases that an individual infected at time *t* would generate if conditions remained as they were at time *t* [33]. The interpretation of case numbers across countries during the pandemic was challenging because of differences in testing policies etc. Since the ascertainment of deaths is less likely to vary across countries and over time, we used the daily reported number of deaths to estimate transmissibility. Thus, we implicitly assumed that at any time, a proportion of cases result in deaths so that the reported number of deaths is a proxy for the cases in a country.

We developed three different models, each of which estimated transmissibility in the recent past and produced forecasts of COVID-19 deaths (Sec. 2.1 to 2.3 in S1 File). Each model was calibrated using the daily reported incidence of deaths and/or cases. For each week, we combined the outputs of these models to build an unweighted ensemble (Sec. 2.4 in S1 File). We produced short-term forecasts (i.e. 1-week ahead), for which changes in the population immunity level could be ignored. Over the course of the epidemic, the effect of the potential depletion of the susceptible population on the trajectory of the epidemic may become more pronounced. Inherently, by estimating transmissibility in real-time, our models account for any general decrease in the proportion of population being susceptible. However, the forecasts produced do not account for any further decrease in this proportion, which may become substantial when forecasting over a medium- to long-term time horizon.

**Fig 1:**
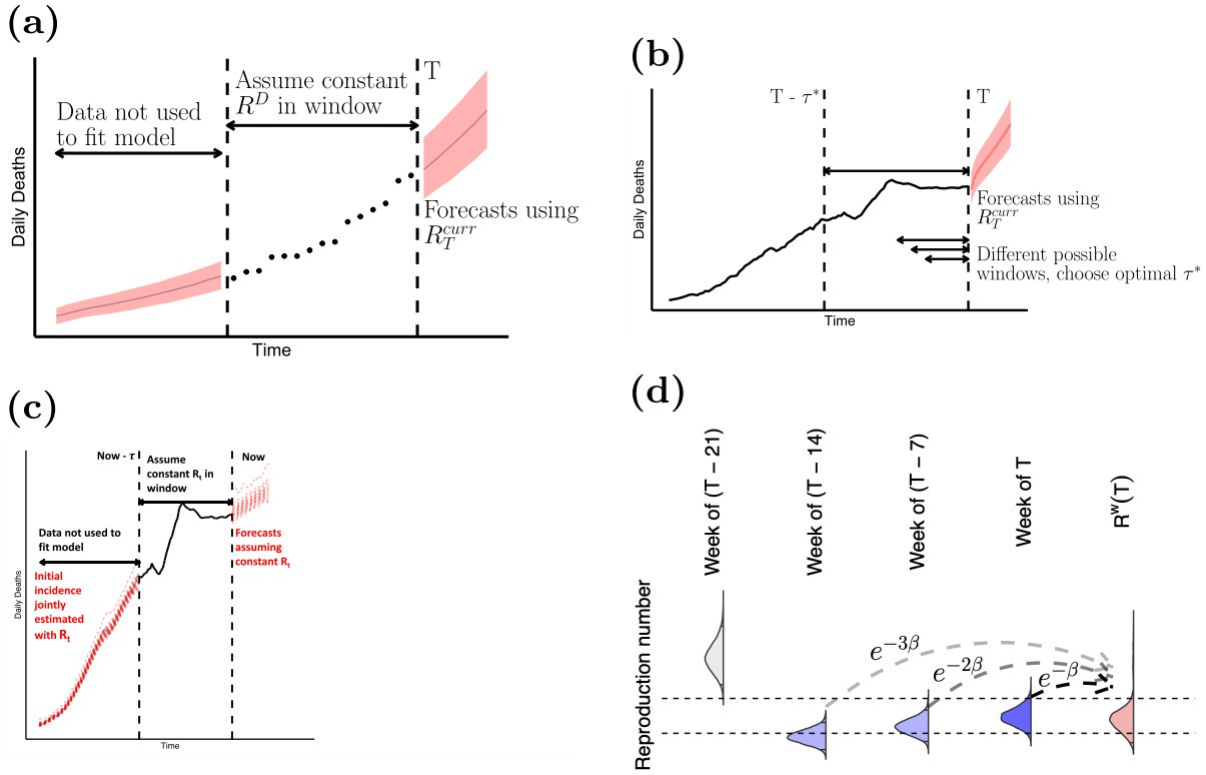
Schematic of the models. (a) Model 1 assumes a single value for $R_{}^{}\left[T-\tau +1,T\right]$. The model is fitted using only the data in this window (*T* − *τ* + 1 to *T*) to jointly estimate the initial incidence of deaths and $R_{}^{}\left[T-\tau +1,T\right]$. For details, see Sec. 2.1 in S1 File. (b) Model 2 optimises the window over which $R_{t}^{}$ is assumed to be constant by minimising the cumulative predictive error over the entire epidemic time series. Estimates from $R_{}^{}\left[T-\tau^{*}+1,T\right]$ are used to forecast into the future, with τ* the window of optimal length. See also Sec. 2.2 in S1 File. (c) Model 3 uses data from both cases and deaths (Sec. 2.3 in S1 File). The dashed blue curve represents the incidence of reported cases weighted by the case-report to death delay distribution, where *μ* is the mean delay. *ρ*_*t*_ is the ratio of the observed deaths and the weighted cases at time *t* and is analogous to an observed case fatality ratio. Forecasts of deaths are obtained by sampling from a binomial distribution using the most recent estimate of *ρ*_*T*_. See also Fig. 3 in S1 File. (d) To obtain medium-term forecasts, we combine the most recent transmissibility estimate $R_{T}^{curr}$ (shown in dark blue) with estimates of transmissibility in the previous weeks to produce a weighted estimate of transmissibility $R_{T}^{w}$ (filled in pink) at time *T*. Estimates from previous weeks are combined with the most recent estimates if the 95% CrI of estimates in week *k*, $R_{T-7k}^{curr}$ overlaps the 95% CrI of $R_{T}^{curr}$. Estimates for weeks where the 95% CrI overlap are shown in light purple, and where the 95% CrI do not overlap in grey. The dashed horizontal lines represent the 2.5^th^ and 97.5^th^ quantile of $R_{T}^{curr}$. We combine the estimates by sampling from the posterior distribution of $R_{T-7k}^{curr}$ with probability proportional to *e*^−β*k^, where *β* is a rate at which the probability decays as we go back in time.

We also produced medium-term forecasts (up to 4-weeks ahead) accounting for the depletion of the susceptible population due to the increased levels of natural host immunity. In order to estimate transmissibility for medium-term forecasts, we combined past estimates of transmissibility into a single weighted estimate $R_{t}^{w}$ as follows. Let *T* denote the last time point in the existing incidence time series of cases or deaths and let $R_{T}^{curr}$ refer to the the most recent estimate of reproduction number for a given model. Starting with the transmissibility estimates of $R_{T}^{curr}$ from the ensemble model, we went back one week at a time, for as long as the 95% credible interval (CrI) of $R_{T^{\prime }}^{curr}$ (where *T*′ < *T*) overlapped the 95% CrI of $R_{T}^{curr}$. We then sampled from the posterior distribution of $R_{T^{\prime }}^{curr}$ in each of those weeks, with a probability that decays exponentially in the past to favour the more recent estimates (Fig 1d). Each week, the rate of decay *β* was optimised by minimising the relative error in the predictions for the previous week.

Since our heuristic for obtaining weighted reproduction number $R_{t}^{w}$ accounts for the stability of the epidemic trajectory in a country, it resulted in the inclusion of a variable number of weekly estimates for each country and each week. For instance, if the transmission levels in a country had been consistent for a long period, weekly estimates of reproduction number from a greater number of weeks contributed to the weighted reproduction number than if the transmission levels had changed in the recent weeks.

As the weighted reproduction number $R_{t}^{w}$ already accounts for the population immunity at time *t*, we first estimated an effective reproduction number defined as the reproduction number if the entire population were susceptible (Eq. 10 in S1 File). We then estimated the reproduction number $R_{t}^{S}$ accounting for the effect of population immunity at time *t* due to infection (Eq. 11 in S1 File).

### Forecast horizon

The short-term forecast horizon was set to be 1 week. We produced forecasts for the week ahead (Monday to Sunday) using the latest data up to (and including) Sunday. We did not model the potential changes in the population immunity levels as any such change is not expected to affect the trajectory of the epidemic over this short time horizon.

The medium-term forecasts were made over a 4-week horizon using $R_{t}^{S}$. Since estimates of the weighted reproduction number could only be obtained once we had sufficient weekly estimates to combine, medium-term forecasts were produced from 29^th^ March to 29^th^ November 2020.

## Epidemic phase

Adapting the categorisation by Abbott et al. [34], we categorised the broad epidemic trends in each country into epidemic phases using the estimated reproduction numbers. Epidemic phases were defined prospectively using $R_{T}^{S}$, and retrospectively using $R_{T}^{curr}$. That is, $R_{T}^{curr}$ was used to define the phase for the most recent week for which we had data.

For the week ending at time *T*, we defined the epidemic phase in a country to be:

- ‘definitely growing’ if $R_{T}^{curr}<1$ in less than 5% of the samples of the posterior distribution;
- ‘likely growing’ if $R_{T}^{curr}<1$ in between 5% and 25% of the samples of the posterior distribution;
- ‘definitely decreasing’ if $R_{T}^{curr}>1$ in less than 5% of the samples of the posterior distribution;
- ‘likely decreasing’ if $R_{T}^{curr}>1$ in between 5% and 25% of the samples of the posterior distribution;

If 25–75% of the samples of the posterior distribution of $R_{T}^{curr}$ were less than 1, we used the uncertainty of the estimates to classify the phase. If the width of the 95% CrI was less than 0.5, we classified the phase as ‘likely stable’, otherwise we deemed it ‘indeterminate’.

When defining the phase prospectively, we used $R_{T}^{S}$ instead of $R_{T}^{curr}$ for the classification. Since $R_{T}^{S}$ was updated for each day of the forecast horizon, we first assigned an epidemic phase to each day using the classification scheme above. We then used the most often assigned phase in a week to define the weekly phase.

### Assessing model performance

The model forecasts were validated against observed deaths as these became available. To quantitatively assess the performance of the model for both short- and medium-term forecasts, we used the following metrics:

- **Mean relative error** The mean relative error (MRE) is a widely used measure of model accuracy [35]. MRE for the forecasts ${\hat{D}}_{t}$ at time *t* is defined as:
  $$MRE_{t}\left(D_{t},{\hat{D}}_{t}\right)=\frac{\sum_{s=1}^{N}\left|D_{t}-{\hat{D}}_{t,s}\right|}{N*(D_{t}+1)},$$
  where *D*_*t*_ denotes the observed deaths at time *t*, *N* is the number of simulated trajectories and ${\hat{D}}_{t,s}$ denotes the *s*^*th*^ simulation at time *t* [36]. That is, MRE at time *t* is the error in forecasts averaged across all simulated trajectories and normalised by the observed incidence. We add 1 to the observed value to prevent division by 0. A MRE value of *k* means that the average error is *k* times the observed value. MRE will be 0 for a perfect model; it should be as small as possible.
- **Comparison with null model** Ratio of the absolute error made by the model with the absolute error made by a null model that uses the average of the last 10 observations as the forecast for the week ahead. We also compared the model error with the error made by a linear model (forecasts from a line fitted to the last 10 observations). A ratio greater than 1 indicates that the model error was larger than that made by the null model i.e., the model performed no better than the null model.
- **Coverage probability** Coverage probability refers to the proportion of observations that are contained in given credible interval (CrI) of the distribution of forecasts. For a well-calibrated model, 50% of the observations should be contained in the 50% CrI [37] (analogous criterion applies to other CrI) i.e., coverage probability should be 0.5. For a X% CrI, coverage probability higher than X% indicates that the model is under-confident with wide CrIs. Similarly, a value less than X% suggests that the model is over-confident with narrow CrIs.

A direct comparison of daily forecasts, which are smooth by definition, to daily data which are noisy can be potentially be misleading. At the same time however, comparing weekly forecasts to aggregated weekly data can lead to artifically lower error through over-smoothing. Therefore we first smoothed the time series of observed deaths for each country and for each week by taking a 3-day rolling mean. The average of the daily MRE was used as the weekly MRE.

## Data

We used the daily number of COVID-19 cases and deaths reported by the World Health Organisation (WHO) [1]. Any data anomalies were corrected using data published by the European Centre for Disease Prevention and Control [38], or other sources (Sec. 4 in S1 File). All data used in the study are available at the github repository associated with this article (https://github.com/mrc-ide/covid19-forecasts-orderly).

## Results

Methods for estimating transmissibility during epidemics typically rely on the time series of incident cases combined with the natural history parameters of the pathogen [39, 40]. However, in the current pandemic, interpretation and comparison of estimates across countries based on the number of cases was made difficult by the differences in case definitions, testing regimes, and variable reporting across countries as well as over time within each country [41]. We therefore developed three different models that relied on the number of reported deaths to estimate COVID-19 transmissibility and to produce short- and medium-term ensemble forecasts of deaths (1- and 4- week ahead respectively). The methods underlying the individual models are illustrated in Fig 1a to 1c (see Methods and Sec. 2 in S1 File for details).

Beginning 8^th^ March 2020, we produced weekly forecasts for every country with evidence of sustained transmission. As the pandemic rapidly spread across the world, the number of countries included in the weekly analysis grew from 3 in the first week (week beginning 8^th^ March 2020), to 94 in the last week of analysis included in this study (week beginning 29^th^ November 2020) (Fig. 1 in S1 File). Our results are based on the analysis done for 81 countries (see Sec. 4 in S1 File for exclusion criterion) over the 39 week period from 8^th^ March to 29^th^ November 2020.

### Short-term forecasts and model performance

**Fig 2:**
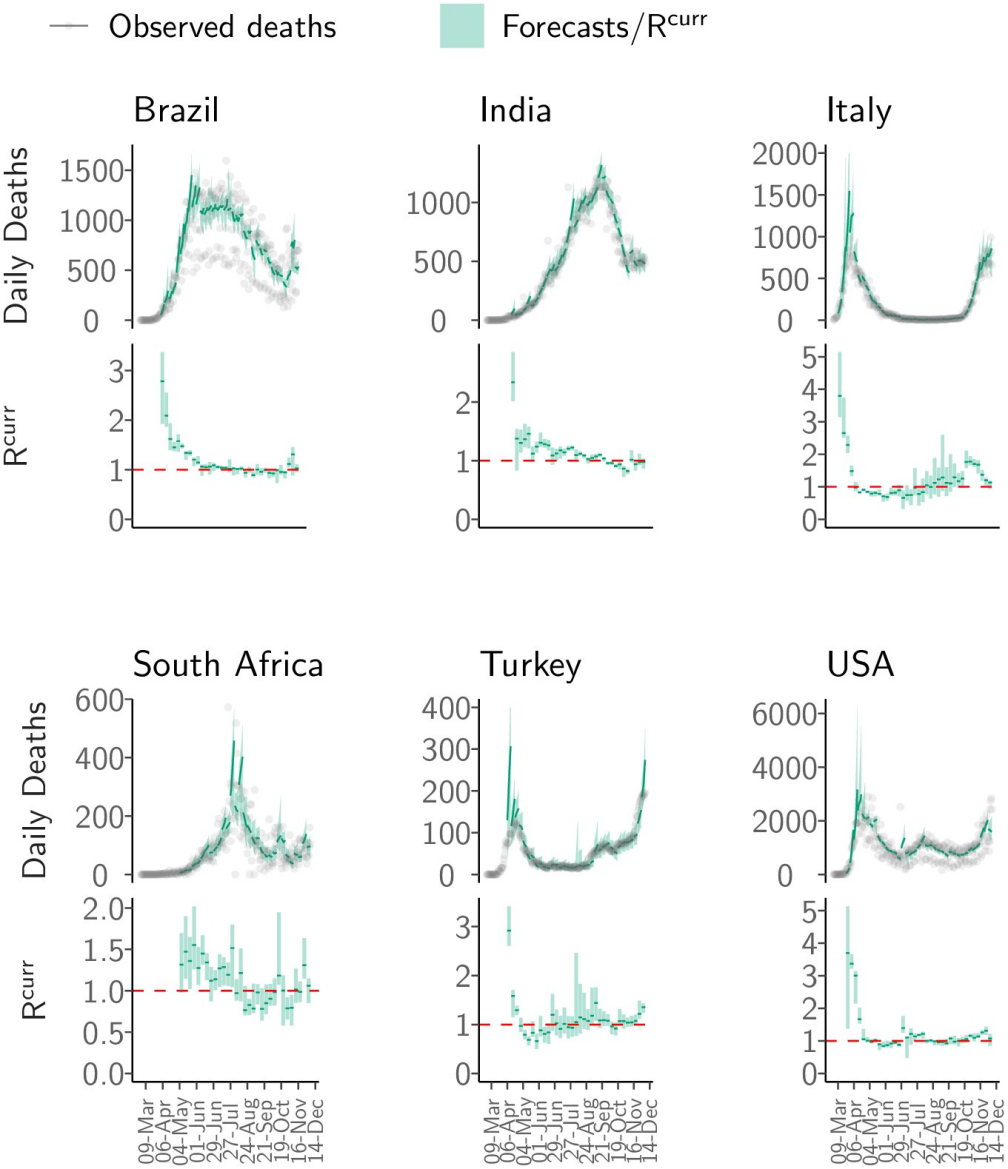
Short-term forecasts. The short-term forecasts and observed deaths for six countries: Brazil, India, Italy, South Africa, Turkey and the United States of America (USA). For each country, the top panel shows the observed deaths in gray; the solid green line shows the median forecast. The shaded interval represents the 95% CrI of forecasts. The forecasts were produced using the most recent estimates of $R_{T}^{curr}$ assuming that the transmissibility remains constant. The bottom panel for each country shows the effective reproduction number ($R_{T}^{curr}$) used to produce the forecasts. The solid green line in the bottom panel for each country is the median estimate of $R_{T}^{curr}$ while the shaded region represents the 95% CrI. The dashed red line indicates the $R_{T}^{curr}=1$ threshold. Note that the y-axis is different for each subfigure. See Fig. 3 in S1 File for results for all other countries.

Overall, the ensemble model performed well in capturing the short-term trajectory of the epidemic in each country (Fig 2). Across all weeks of forecast and all countries, an average 58.7% (SD 32.4%) of the observations were in the 50% credible interval (CrI) and 89.4% (SD 21.7%) of the observations were in 95% CrI (for a breakdown by country and week of forecast see Sec. 7.5 in S1 File).

**Fig 3:**
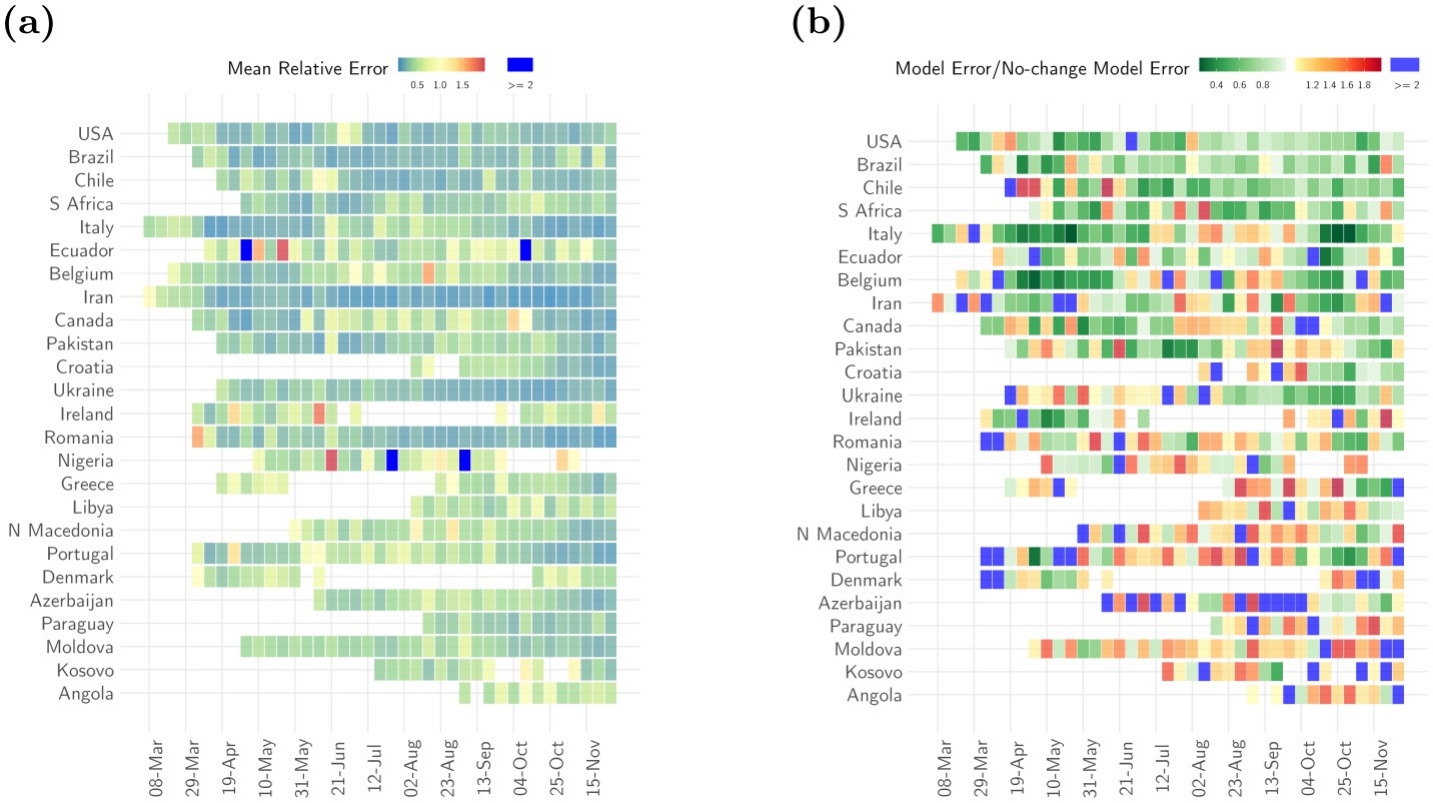
Short-term forecasts MRE and comparison with null model. (a) The mean relative error of the ensemble model for each week of forecast (x-axis) and for each country (y-axis). Dark blue cells indicate weeks where the relative error of the model was greater than 2. (b) The ratio of the absolute error of the model to the absolute error of a no-change null model that uses the average of the last 10 days as a forecast for the week ahead. Shades of green show weeks for a given country where the ratio was smaller than 1 i.e., the ensemble model error was smaller, and weeks where the ratio was greater than 1 i.e. the ensemble model error was larger than the null model error are shown in shades of red (yellow to red). Dark blue indicates weeks when the ratio was larger than 2. In order to present a representative sample, we first ranked all countries by the percentage of weeks in which ensemble model error was smaller than the null model error. We then selected every third country from the top 75 countries in this list. Results for the selected 25 countries are shown here. See Fig. 4 in S1 File for the results for other countries. Ordering of countries in the figure reflects the order in the ranked list i.e. countries with the highest percentage of weeks with model error smaller than null model error are shown on the top.

The mean relative error (MRE) across all countries and all weeks was 0.4 (SD 0.4) (Fig 3). That is, on average the model forecasts were 0.4 times lower or higher than the observed incidence. In most countries, the reporting of both cases and deaths through the week was variable, with fewer numbers reported on some days of the week (typically, Saturday and Sunday). The variability in reported deaths strongly influenced the model performance. The MRE scaled linearly with the coefficient of variation (ratio of the standard deviation to the mean) in the reported deaths for the week for which forecasts were made. Thus, the error in forecasts was on average similar to the variability in the reported deaths (Fig. 6 in S1 File). The MRE of the model scaled inversely with the weekly incidence i.e. the error was relatively large when the incidence was low (Fig. 6 in S1 File). This might reflect that either small absolute errors translate into large relative error when observed values are small, or inherently more unstable estimates of reproduction number when the incidence is low [42], or a combination of these factors.

The model performance was largely consistent across epidemic phases (growing, likely growing, decreasing, likely decreasing, likely stable, and indeterminate with similar coverage probability and MRE (Table 1 in S1 File). The slightly larger proportion of observations in the 50% and 95% credible intervals for the ‘indeterminate’ phase and the larger MRE in this phase together suggest that the model was ‘under-confident’ with large credible intervals [43].

We compared the performance of the model with that of a null no-change model. In most instances, the ensemble model outperformed the null model. In 76.8% of the weeks in ‘definitely decreasing’ phase and 74.4% of weeks in ‘definitely growing’ phase, the absolute error of the model was smaller than the error made by the null model (Fig 3, Sec. 7.2 and Table 2 in S1 File). The null model performed better when the trajectory of the epidemic in a country was relatively stable exhibiting little to no change over the time frame of comparison. This is to be expected as the null model describes precisely this stable dynamic. Indeed, in 69.3% of the weeks in the ‘likely growing’ phase and 75.3% weeks classified as ‘indeterminate’ phase, the absolute error of the model was larger than the error made by the null model. However, the relative error of the model remained small (MRE 0.50, SD 0.62) even in countries and weeks where it did not perform as well as the null model. Similarly, our model performed better than a linear growth model across all phases, specifically in 90.8% of the weeks in ‘definitely decreasing’ phase and 79.6% weeks in ‘definitely growing’ phase (Sec. 7.3, Table 2 in S1 File).

### Medium-term forecasts and model performance

**Fig 4:**
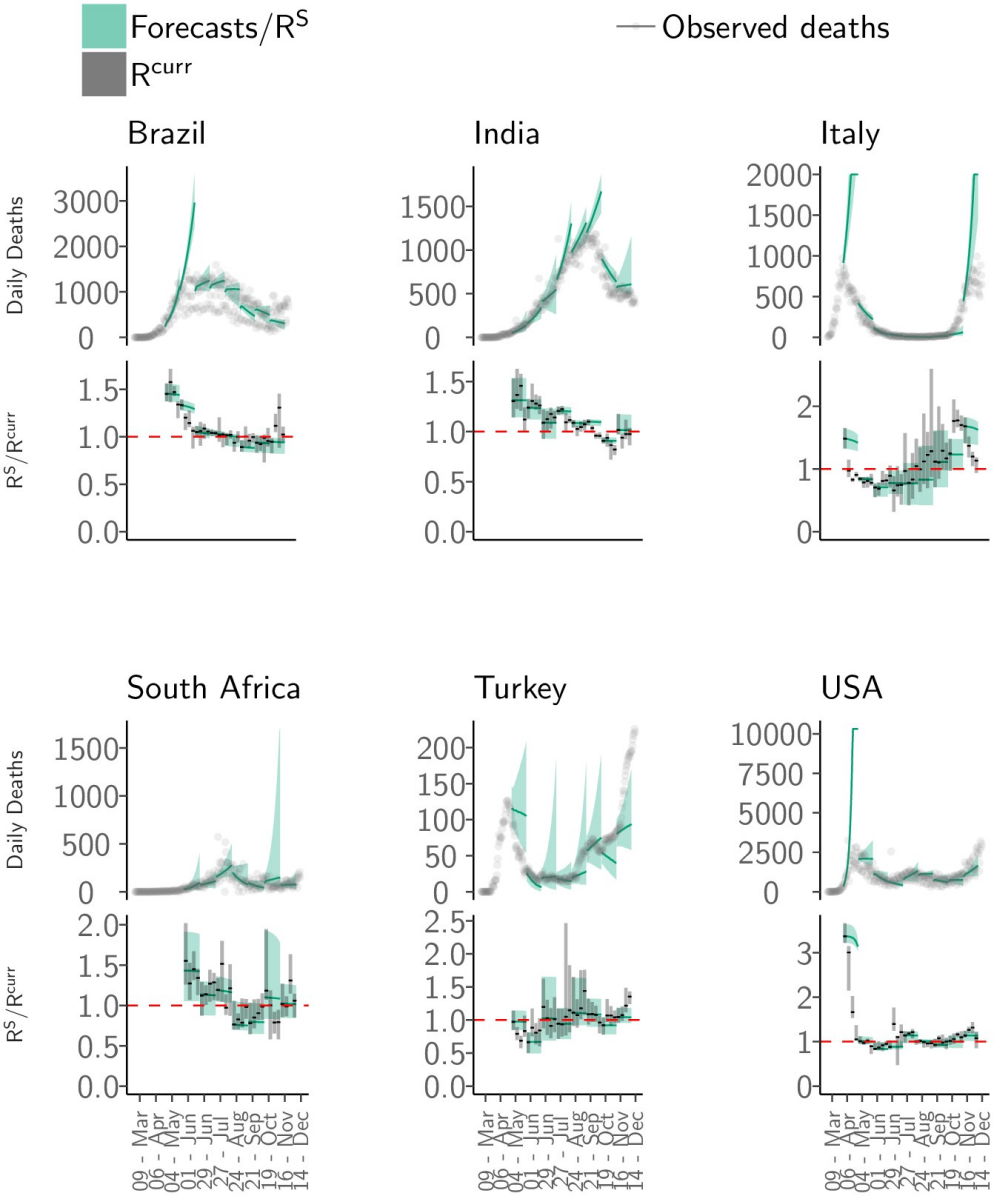
Medium-term forecasts. The medium-term forecasts and observed deaths for six countries: Brazil, India, Italy, South Africa, Turkey and the United States of America (USA). For each country, the top panel shows the observed deaths in grey; the solid green line shows the median the 4-weeks ahead forecast. The shaded interval represents the 95% CrI of forecasts. The bottom panel for each country shows the median (solid black line) and the 95% CrI (grey shaded area) of weekly estimate of $R_{t}^{curr}$ from the short-term forecasts and the median (green line) and the 95% CrI (shaded green area) of $R_{t}^{S}$ i.e. the reproduction number accounting for depletion of susceptible population from the medium-term forecasts over a 4-week horizon (Methods). The dashed red line indicates the $R_{t}^{S}=1$ threshold. Note that the y-axis is different for each subfigure. The forecasts were produced every week over a 4-week forecast horizon. The figure shows all non-overlapping forecasts over the course of the pandemic. See Fig. 3 in S1 File for results for all other countries and weeks.

The rapidly changing situation and the various interventions deployed to stem the growth of the pandemic make forecasting at any but the shortest of time horizons extremely challenging [44, 45]. Despite these challenges, we found that our medium-term forecasts robustly captured the epidemic trajectory (Fig 4) in all countries included in the analysis (Fig 4).

**Fig 5:**
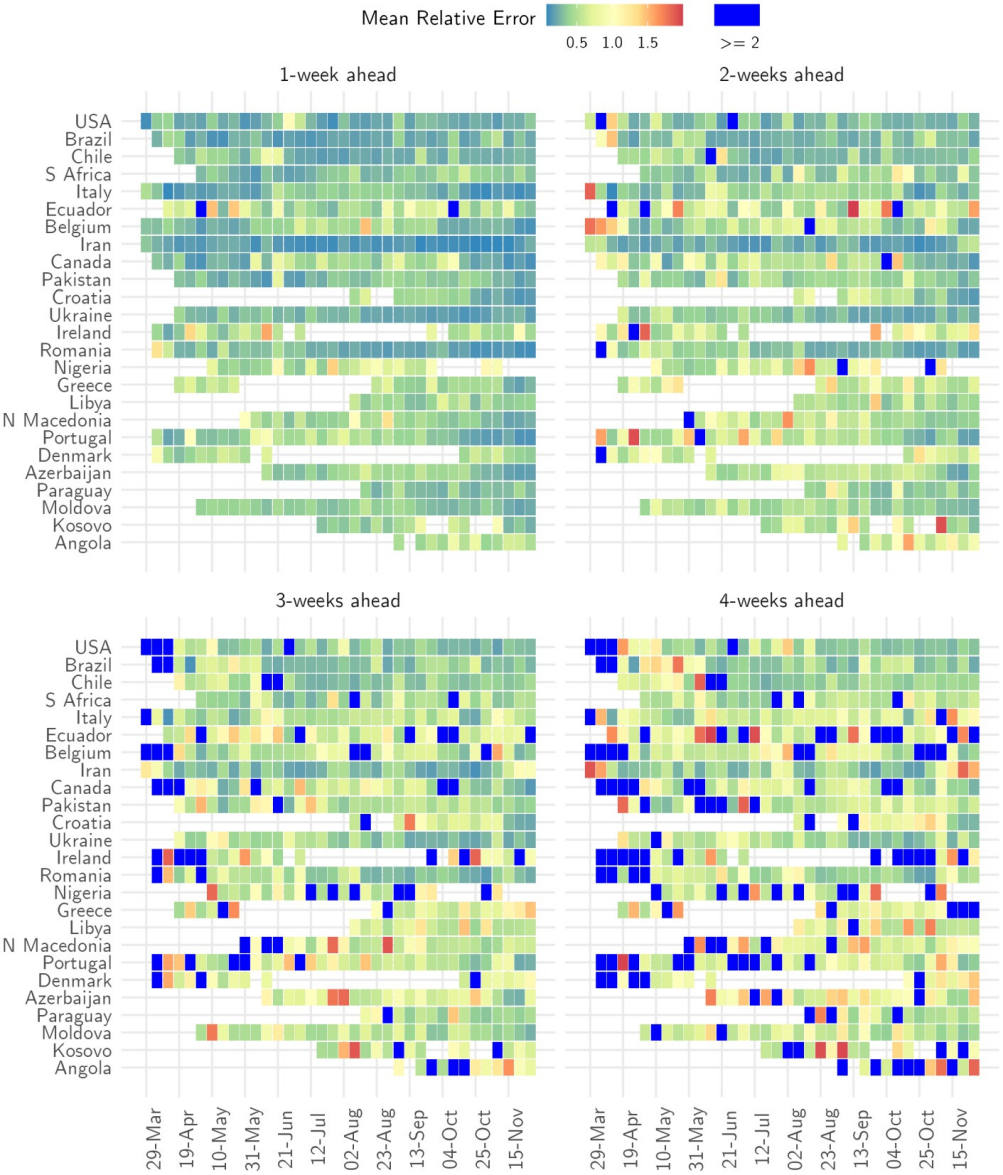
Relative error of medium-term forecasts. The mean relative error of the model in 1-week, 2-week, 3-week and 4-week ahead forecasts for each week when a forecast was made (x-axis) and for each country (y-axis). Blue cells indicate weeks where the relative error of the model was greater than 2. For ease of presentation, results are shown for the same 25 countries as Fig 2. See Sec. 7 in S1 File for the results for other countries.

Overall, the MRE remained small over a 4-week forecast horizon, with errors increasing over the projection horizon (Sec. 8.1 in S1 File). We therefore restricted the projection horizon to 4 weeks. The MRE across all countries in 1-week ahead forecasts was 0.4 (SD 0.3), increasing to 2.6 (SD 28.3) in 4-week ahead forecasts (Fig 5, Fig. 10 in S1 File). The MRE for 1-week ahead forecasts was less than 1 (indicating that the magnitude of the error was smaller than the observation) in 91.1% of weeks for which we produced forecasts. The corresponding figure for 4-week ahead forecasts was 66.0% (Table 3 in S1 File).

The proportion of observations in the 50% CrI remained consistent across the forecast horizon and varied from 56.3% (SD 33.4%) in 1-week ahead forecasts to 45.6% (SD 40.9%) in 4-week ahead forecasts (Figs. 11 and 12 in S1 File).

**Fig 6:**
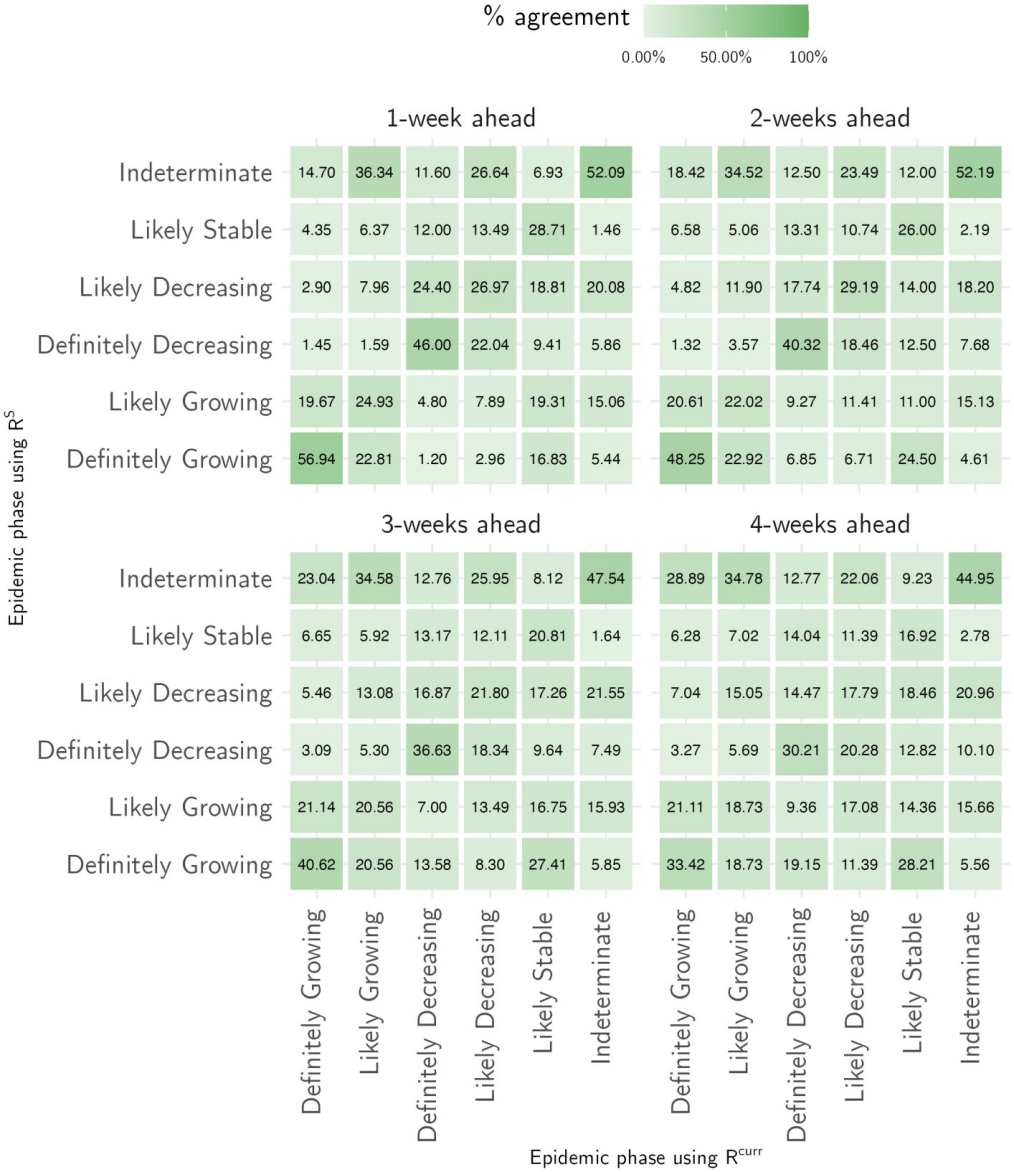
Characterisation of the epidemic phase. For a given retrospective classification of epidemic phase using the weekly estimates of the reproduction number from the short-term forecasts(x-axis), the figures in the cell show the percentage of weeks for which the prospective characterisation was consistent using the medium-term reproduction number estimates (show on the y-axis).

Across the 81 countries for which we produced both short- and medium-term forecasts, the epidemic phase estimated prospectively using the reproduction number estimates from medium-term forecasts, $R_{T:T+28}^{S}$ (Sec. 3 and Eq. 11 in S1 File), was consistent with the retrospective phase assigned using the estimates from the short-term forecasts ($R_{T}^{curr}$) in 41.7% (873/2094) weeks in 1-week ahead forecasts and in 28.9% (521/1804) weeks in 4-week ahead forecasts (Fig 6). When the phase definitions using $R_{T:T+28}^{S}$ and $R_{T}^{curr}$ were different, the medium-term estimates most frequently misclassified them as a phase that had greater uncertainty. For instance, in 601 weeks when the epidemic phase was identified as ‘definitely decreasing’ using weekly estimates and incorrectly characterised using medium-term estimates, it was misclassified as ‘likely decreasing’, ‘likely stable’ or ‘indeterminate’ in 71.3% (429/601) weeks. Similarly, in the misclassified weeks, when the epidemic phase using weekly transmissibility estimates was ‘definitely growing’, the medium-term classification was ‘indeterminate’ in 38.3% (367/959) and ‘likely growing’ in 37.8% (362/959) weeks. This mis-characterisation is expected as the uncertainty in estimates of $R_{T:T+28}^{S}$ grows over the forecast horizon. Crucially, in the weeks where the epidemic phase was misclassified using $R_{T:T+28}^{S}$, the prospective classification indicated the opposite trend (growing classified as decreasing or vice versa) in only 14.8% weeks (737/4992). This finding shows that the medium-term transmissibility estimates can be used a reliable indicator of the overall direction of the epidemic trajectory.

## Discussion

Models used to forecast COVID-19 cases and/or deaths vary in complexity in the data used for model calibration. More complex and/or granular models rely on multiple data streams including data on hospital admissions and occupancy, testing, serological surveys and data on patient clinical progression and outcomes [22]. Such complex location-specific models can provide crucial insights into the ongoing epidemic and inform targeted public health interventions by synthesising evidence from different data streams. However, scaling such analysis to include multiple geographies is challenging because of the variability in availability and reliability of local surveillance data. The computational time needed to fit complex models make scaling them difficult and delays the timely provision of risk estimates.

Furthermore, the wide-scale societal and behavioural changes brought about by the pandemic impose practical constraints on utilising data that are available for multiple countries. For instance, widely available data on the changes in mobility inferred from mobile phone usage released by Google and Apple were informative of the changes in transmission in the early phase of the COVID-19 pandemic and were used in several modelling studies [46, 47]. Although these data continue to be available, evidence suggests a decoupling of transmission and mobility in most countries [48, 49]. Models that relied on such additional data or assumptions about non-pharmaceutical interventions [47, 50] could not fit the observed trajectory as the situation continued to change over the course of the epidemic.

Efforts to model and forecast COVID-19 transmission dynamics must therefore meet the challenges of a long and ongoing pandemic spread over an unprecedented scale. Modelling groups around the world have attempted to meet one or both challenges with various analyses conducted at a sub-national scale [51], at a national scale for a specific country [23, 52–54], and for several countries across the globe [34, 55–58]. In contrast to models built for a region or country and calibrated using local data, models that aim to provide a global overview must be sufficiently general to describe the epidemic trajectory in a range of countries/regions using widely available data that are consistently available over time.

We have produced short-term forecasts and estimates of transmissibility for 81 countries for more than 100 weeks at the time of writing, implementing three simple models that use only the time series of COVID-19 cases and deaths. We have thus traded particularity for generality, to allow us to carry out analysis for a large number of countries over a long period of time. Many other efforts that aimed to generate forecasts for multiple countries were discontinued as the situation evolved rapidly [47, 50, 59, 60] (note—the webpages have been discontinued). The simplicity of our methods, which make few assumptions and use routine surveillance data, allowed us to provide weekly updates for the last two years. Our transmission models, analysis pipeline, and the web interface can also be reused to provide similar inputs during future outbreaks.

With the increasing use of infectious disease models and forecasts, model evaluation has also received increasing attention from the research community and a wide variety of metrics for scoring forecasts have been developed [61]. Some of these metrics, such as coverage probability, have well-defined targets that an ideal model should achieve. However, a “good” or “acceptable” range for other commonly used metrics such as absolute error or mean relative error is not always well-defined, and these metrics are typically used to generate a relative ranking of different models [52, 62]. Future research could explore the development of standardised performance indicators and evaluation frameworks for forecasts that are tied to public health objectives. Here we relied on a few key, interpretable metrics to retrospectively evaluate the performance of our model.

Despite the challenges inherent in forecasting a fast-moving pandemic in the presence of unprecedented public health interventions, our ensemble model was able to successfully capture the short-term transmission dynamics across all countries included in the analysis with small relative error in the weekly forecasts across different COVID-19 waves in each country. The variable performance of our model in weeks and countries with fewer deaths and/or large variability in reported deaths over weeks reflects this trade-off. Similarly, in common with most forecasting methods [34, 55], two of the models assumed that transmissibiity remained unchanged over the week for which forecasts were made, which led to large relative errors close to changes in the overall trends (growing to declining, or vice versa). In the absence of more detailed data, we assumed that epidemiological parameters such as the delay from onset of symptoms to death were the same across all countries and throughout the period of analysis. These parameters are likely to vary over time and between countries, and using country-specific parameters could lead to moderate improvements in the model fits and forecast performance.

Due to the variability in testing and reporting of cases across different countries and over time within countries, using the reported number of cases to estimate transmissibility and produce forecasts is difficult without using more complex models. For these reasons, we primarily used deaths to estimate the reproduction number as we assumed that reporting of COVID-19 deaths was more complete and consistent over time and across different country surveillance systems. Although this assumption is unlikely to hold for many countries [63–65], our methods are robust to a constant rate of under-reporting over time as this would not alter the overall epidemic trends. A limitation of our work is that our estimates reflect the epidemiological situation with a delay of approximately 19 days (the delay from an infection to a death [50]). Nevertheless, our short-term forecasts and transmissibility estimates provide valuable global overview and continuous insights into the dynamic trajectory of the epidemic in different countries. They also provide indirect evidence about the effectiveness of public health measures. Future research could investigate integrating more data streams such as local health capacity (e.g., from Healthsites.io) into the models. In addition to the weekly reports that we publish, our work contributed to other international forecasting efforts [23, 43, 52].

We developed a simple heuristic to combine past estimates of transmissibility and a decline in the proportion of susceptible population to produce medium-term forecasts. These forecasts were produced under the assumption that the transmission trends remain unchanged over the forecasting horizon, except for a depletion of susceptible population due to naturally-acquired immunity. In the early phase of the pandemic, transmission dynamics of the pandemic were likely to have been strongly influenced by the stringent interventions that were deployed in several countries leading to rapid changes in transmissibility. Depletion of susceptible population did not substantially affect the transmission trends in this period, especially in countries with large populations. Therefore, model performance in early 2020 was modest with forecasts of very large number of daily deaths for some countries Fig 4. Model performance in forecasting up to 4 weeks ahead was better in late 2020. Consistent with findings from other modelling studies [23], we found that the model error was unacceptably high beyond 4 weeks, suggesting that forecasting beyond this horizon is difficult. Importantly, our prospective characterisation of the epidemic phase using weighted estimates of transmissibility were largely in agreement with the retrospecitve classification using short-term transmissibility estimates. Thus, our method was successful at capturing the broad trends in transmission up to 4 weeks ahead. The medium-term forecasts can therefore serve as a useful planning tool as governments around the world plan further implementation or relaxation of non-pharmaceutical interventions.

Our method incorporates the depletion of susceptible population and hence can in principle be extended to account for increasing population immunity as vaccination is rolled out across the world. However, inclusion of vaccine induced immunity depends on the availability of reliable data on vaccination coverage. Further, even if such data were available, teasing apart the impact of vaccination on transmission and mortality could be non-trivial. In light of these issues, it might be challenging to extend our approach to rigorously assess the effect of vaccination on epidemic trajectory on a global scale. These challenges are further compounded by the emergence of variants of concern with immune evasion characterisitcs such as Omicron, which increase the risk of reinfection. However, our latest estimates of transmissibility indirectly reflect the impact of vaccination on transmission, allowing for the delay from vaccination to full immunity, and from infection to death. As we continue to track COVID-19 transmissibility globally, any temporal changes in transmissibility would implicitly account for the changes due to differential vaccination coverage.

Mathematical modelling and forecasting efforts have supported data-driven decision making throughout this public health crisis. Our aim was to provide a global overview and hence improve situational awareness, and not to provide a new modelling paradigm. We therefore chose to use established models and in addition develop a simple model to realise our objective. Using relatively simple approaches, we produced robust forecasts for COVID-19 in 81 countries and provided crucial and actionable insights. As the world continues to grapple with renewed waves of COVID-19 cases, and against the backdrop of an increasingly complex population immunity landscape, modelling outputs should continue to be evaluated to assess their utility in informing public health response.

## Code

All analysis was carried out in R version 4.0.2. The code for the analysis is available as orderly [66] project at https://github.com/mrc-ide/covid19-forecasts-orderly. DeCa model is available as an R package at https://github.com/sangeetabhatia03/ascertainr. The accompanying R package https://github.com/mrc-ide/rincewind contains utility functions for creating the figures and processing model outputs.

## Supporting information

S1 File Supplementary methods and results. The supplementary file contains a description of the methods and details on data, epidemiological parameters, additional results on model performance.
(ZIP)

S2 File Web tool. An interactive web-tool available at https://shiny.dide.imperial.ac.uk/covid19-forecasts-shiny/ presents both short- and medium-term forecasts, and reproduction number estimates for all countries included in the analysis.
(TXT)
