# Supplementary material for: Retrospective evaluation of real-time estimates of global COVID-19 transmission trends and mortality forecasts
Source: PLoS One. 2023 Oct 18;18(10):e0286199. doi: 10.1371/journal.pone.0286199 (PMC10584190; doi:10.1371/journal.pone.0286199)
Supplement: S1 File — The supplementary file contains a description of the methods and details on data, epidemiological parameters, additional results on model performance. (ZIP) [file pone.0286199.s001.zip › si.pdf]

Supplementary Information for Retrospective evaluation of real-time estimates of global COVID-19 transmission trends and mortality forecasts

Contents

1 Overview **2**

2 Methods **2**

2.1 RtI0 . . . 2

2.2 APEestim . . . 3

2.3 DeCa . . . 4

2.4 Ensemble Model . . . 5

3 Medium-term forecasts **6**

4 Data and epidemiological parameters **6**

4.1 Cleaning and pre-processing steps . . . 7

4.2 Inclusion/Exclusion Criteria . . . 7

5 Infection Fatality Ratio (IFR) **9**

6 Augmentation of observed cases for DeCa **11**

7 Model performance assessment **13**

7.1 Mean relative error by epidemic phase . . . 13

7.2 Relative error and comparison with no-change model . . . 13

7.2.1 Comparison with no-change and linear models by phase . . . 15

7.3 Relative error and comparison with a linear model . . . 15

7.4 Mean relative error compared with the weekly CV . . . 18

7.5 Coverage Probability . . . 18

8 Medium-term forecasts **20**
8.1 Relative error . . . 21
8.2 Coverage Probability . . . 25

1 Overview

This supplementary document presents the methods underlying the individual models for the short-term forecasts SI Sec. 2, and updating the reproduction number for the medium-term forecasts to account for population immunity due to infection SI Sec. 3. We also present details of the data and epidemiological parameters used SI Sec. 4 and the criteria for including/excluding a country from the analysis SI Sec. 4.2. We present additional results on the performance assessment of the model for short- (SI Sec. 7) and medium-term forecasts (SI Sec. 8).

Notation

Hereafter, D_t and C_t represent the number of reported COVID-19 deaths and cases at time t respectively. $R[t_1, t_2]$ is the reproduction number between times t_1 and t_2. The most recent estimate of transmissibility is denoted as R_T^{curr}. We use the serial interval distribution of cases as a proxy for the serial interval distribution of deaths (denoted using ω), noting that the two will have the same mean but different variance akin to the relation between generation time and serial interval distribution [1]. Estimated incidence of deaths at time t is denoted by $\widehat{D}_t$. T refers to last time point in the existing incidence time series of cases or deaths.

2 Methods

2.1 RtI0

The first model relies on a well-established method [2] that assumes the daily incidence of deaths is approximated with a Poisson process following the renewal equation [3]:

$$D_t \sim Poisson\left(R_t \sum_{s=1}^{t} D_{t-s}\omega_s\right) \tag{1}$$

A standard approach to inferring recent transmissibility from an incidence time series relies on the assumption that the effective reproduction number is constant over a window (i.e. the “calibration window”) back in time of size τ time units (for example days or weeks) [4]. Adopting a similar approach here, we estimated R_t using only the data in a fixed time-window (of τ days) prior to the most recent observation to calibrate the model. We estimated the average transmissibility $R[T-\tau+1, T]$ over that time-window, but made no assumptions regarding the epidemiological situation or transmissibility prior to this calibration window. Instead, we jointly estimated (using Markov Chain Monte Carlo (MCMC)) combinations of $R[T-\tau+1, T]$ and the incidence of deaths prior to the calibration window $\widehat{D}_t$ for $t = \{1, 2, \ldots T-\tau\}$ that are consistent with the observed deaths in the time window $[T-\tau+1, T]$.

The model likelihood is given by

$$\begin{aligned}
&\mathcal{L}\left(\left\langle \widehat{D}_t \right\rangle, R[T-\tau+1,T] \mid D_{T-\tau+1},\ldots,D_T\right)\\
&= \prod_{s=T-\tau+1}^{T} P\left(D_s \mid \left\langle \widehat{D}_t \right\rangle, R[T-\tau+1,T], D_{T-\tau+1},\ldots D_{s-1},\right)\\
&= \prod_{s=T-\tau+1}^{T} Poisson\left(D_s \mid R[T-\tau+1,T]\sum_{k=1}^{s} D_{s-k}\omega_k\right)
\end{aligned} \tag{2}$$

where $\left\langle \hat{D}_t \right\rangle = \{D_1, D_2, \ldots D_{T-\tau}\}$ and $\widehat{D}_t = D_t$ for $t = T-\tau+1,\ldots,T$.

The most recent estimate of transmissibility R_T^{curr} in this model is $R[T-\tau+1,T]$. We then sampled sets of back-calculated early incidence time series $(\widehat{D}_1,\ldots \widehat{D}_{T-\tau})$ and reproduction numbers $(R[T-\tau+1,T])$ from the joint posterior distribution obtained in the estimation process, and projected future incidence $\widehat{D}_{T+i}$ for $i \geq 1$ conditional on these as follows:

$$\begin{aligned}
\widehat{D}_{T+i} \sim Poisson\Bigg(R_T^{curr} \sum_{k=1}^{T+i-1} D_{T+i-k}\omega_k \,|R_T^{curr}, \widehat{D}_1,\ldots \widehat{D}_{T-\tau}, D_{T-\tau+1},\ldots D_T,\\
\widehat{D}_{T+1},\ldots,\widehat{D}_{T+i-1}\Bigg),
\end{aligned} \tag{3}$$

where $\widehat{D}_t = D_t$ for $t = T-\tau+1,\ldots,T$.

During the period covered in the analysis, the epidemiological situation in most countries was changing rapidly with public health measures being reviewed weekly. At the same time, there was a strong 'weekend effect' in the observed data, with typically fewer deaths reported on Saturdays and Sundays. We therefore assumed a fixed calibration window of 10 days to incorporate the rapid dynamics and offset the lower reporting over the weekend. We ran the MCMC for 10000 iterations. We sampled 1000 sets of R_T^{curr} and back-calculated incidence, and for each sampled set, we drew 10 stochastic realisations of the projected incidence of deaths.

2.2 APEestim

Similarly to Model 1, Model 2 relies on the renewal equation (SI Eq. 1) but uses the full time series of observed deaths, and uses information theory to optimise the choice of the calibration window i.e. the time-window of size τ over which $R[T-\tau+1,T]$ is assumed to be constant in the estimation process [5]. Choices of window size can influence the bias and variance of resulting estimates of transmissibility [6]. We integrated over the entire posterior distribution of R_t (under a given window size), to obtain the posterior predictive distribution of incidence at time $t+1$ as

$$P\left(D_{t+1} \mid D_1, D_2, \ldots D_t\right) = \sum_{\mathcal{R}[t-\tau+1,t]} P\left(D_{t+1} \mid D_1, D_2, \ldots D_t, R_t\right) \tag{4}$$

where the sum is over samples from the posterior distribution of R_t τ $\mathcal{R}[t-\tau+1,t]$ assuming a window of

length . We computed this distribution sequentially for $t \in \{1, 2, \dots T-1\}$ and then evaluated every observed count of deaths according to their likelihood under the posterior predictive distribution. This allowed us to construct the accumulated predictive error (APE) for a window length τ and under a given serial interval distribution as [5]:

$$APE_\tau = \sum_{t=1}^{T-1} -\log P\left(D_{t+1} \mid D_1, D_2, \dots D_t\right) \tag{5}$$

Here, D_{t+1} is the observed number of deaths at time $t+1$. The optimal window length τ^* was then chosen as the window for which APE_τ is minimised, optimising the bias-variance trade-off (long windows reduce the estimate variance but increase bias and short windows do the converse).

Again, forward projections were made assuming that transmissibility over the projection horizon remains the same as that in the last τ^* days. That is, R_T^{curr} is set to be $R[T-\tau^*+1, T]$. We then obtain forecasts of deaths as

$$\widehat{D}_{T+i} \sim Poisson\Bigg(R_T^{curr} \sum_{k=1}^{T+i-1} D_{T+i-k}\omega_k \mid D_1, \dots, D_T, \widehat{D}_{T+1}, \dots \widehat{D}_{T+i-1},\Bigg), \tag{6}$$

for $i \geq 1$. We drew 1000 samples from the posterior distribution of R_T^{curr} and for each sampled value, simulated 10 forward trajectories.

2.3 DeCa

Models 1 (RtI0) and 2 (APEestim) use only the time series of deaths to estimate R_t. Model 3 exploits the signal from both the reported deaths and cases to forecast deaths. We assumed that the delay δ between a case being reported and the case dying (for those who die) is distributed according to a gamma distribution with mean μ and standard deviation σ. Let f_Γ be the probability mass function of a discretised gamma distribution. The cumulative number of reported cases at time t weighted by the delay distribution from case report to death, $\sum_0^\infty f_\Gamma(x \mid \mu, \sigma) C_{t-x}$, represents the potential number of deaths at time t, if all cases were to die. The ratio ρ_t of the observed number of deaths to this quantity at time t can be thought of as an observed case fatality ratio. We assume that deaths are distributed according to a binomial distribution:

$$D_t \sim Binomial\left(\sum_0^\infty f_\Gamma(x \mid \mu, \sigma) C_{t-x}, \rho_t\right). \tag{7}$$

The model likelihood is given by

$$
\begin{aligned}
&\mathcal{L}\left(\rho_1, \rho_2, \ldots, \rho_T \mid C_1, \ldots, C_T, D_1, D_2 \ldots D_T, \mu, \sigma\right) \\
&= \prod_{s=1}^{T} P\left(D_s \mid C_1, \ldots, C_s, \rho_s, \mu, \sigma\right) \\
&= \prod_{s=1}^{T} Binomial\left(\sum_{0}^{\infty} f_{\Gamma}(x \mid \mu, \sigma) C_{t-x}, \rho_t \mid C_1, \ldots C_s, \rho s, \mu, \sigma\right).
\end{aligned} \tag{8}
$$

We obtained a posterior distribution for $\rho_1, \rho_2, \ldots, \rho_T$ using the conjugate beta prior for ρ_t (using the R package binom [7]), assuming that parameters of the delay distribution μ and σ are known and fixed. The forecasted number of deaths at time $T+i$ for $i \geq 1$ were then drawn from a binomial distribution as

$$
\widehat{D}_{T+i} \sim Binomial\left(\sum_{k=0}^{T+i-1} f_{\Gamma}(k \mid \mu, \sigma) C_{T+i-k}, \rho_T\right). \tag{9}
$$

Note that the number of deaths at time $T+i$ depends on the number of cases from the beginning of the epidemic to time $T+i$ for $i \geq 1$. That is, for forecasting deaths at time $T+i$, we need the number of cases at time $t > T$. To augment the observed time series of cases, we assumed that the cases in beyond T are distributed according to a gamma distribution with mean and standard deviation of the observed cases in the last week, implicitly assuming no growth or decline in cases. We assessed the extent to which this assumption affected our results (SI Sec. 6). Finally, to include transmissibility estimates from this model in the ensemble, we estimated R_T^{curr} using the observed and median forecasted deaths $D_1, \ldots D_T, \widehat{D}_{T+i}$ for $i \geq 1$. using the R package EpiEstim [4].

We drew 10000 samples from the posterior distribution of ρ_T and 10000 samples from a gamma distribution to augment the observed cases. We then drew 10000 samples from a binomial distribution (eq. (9)) for each pair of augmented cases trajectory and sampled ρ_T.

2.4 Ensemble Model

For each week, we combined the estimates of R_T^{curr}, and the outputs of models RtI0, APEestim, and DeCa into an unweighted ensemble model by sampling the forecasts and reproduction number from each model described above. We also explored building a weighted ensemble by weighting the contribution of each model according to the relative error of the model in the previous week, all previous weeks, across all countries, or estimating the weights independently for each country. We did not find any substantial difference in the performance of the unweighted and weighted ensembles (not shown here). We therefore restricted our analyses to an unweighted ensemble model.

We first drew 10000 samples from the posterior distribution of R_T^{curr} and forecasts from each model and then sampled each posterior distribution with equal weight to build the ensemble posterior distribution of R_T^{curr} and corresponding forecasts.

3 Medium-term forecasts

Accounting for depletion of the susceptible population due to naturally-acquired immunity

As the weighted reproduction number R_t^w already accounts for the population immunity at time t, we first estimated an effective reproduction number R_t^{eff}, defined as the reproduction number if the entire population were susceptible. That is,

$$R_t^{eff} = \frac{R_t^w}{p_t^S} \tag{10}$$

where R_t^w is the weighted reproduction number at time t and p_t^S is the proportion of population that is susceptible to infection at time t. p_t^S is given by $1 - \sum_{j=0}^{t} I_j/N$ where I_j is the number of infections at time j and N is the total population. In estimating the potential future population immunity using this formulation, we only accounted for naturally acquired immunity assuming that the immunity acquired after infection persists. Since we were forecasting deaths (rather than infections), the true number of infections was estimated using a country-specific age-distribution weighted Infection Fatality Ratio (IFR) (SI Sec. 5).

We then incorporated the effect of a declining proportion of susceptible population due to naturally acquired immunity as

$$R_{t+i}^S = R_t^{eff} p_{t+i}^S \tag{11}$$

From the ensemble estimates of R_T^{curr}, we first estimated R_T^S. The medium-term forecasts were then produced using the renewal equation (SI Eq. 1) and the forecasts used to update the estimates of R_{T+i}^S for each $i \geq 1$ over the forecast horizon. We use the notation $R_{t:t+i}^S$ to denote the set of transmissibility estimates from day t to $t+i$ accounting for the depletion of susceptible population.

4 Data and epidemiological parameters

For the weekly analysis, we defined a country as having evidence of active transmission if at least 100 deaths had been reported, and at least ten deaths were observed in each of the past two weeks. Some countries were excluded from the analysis despite meeting these thresholds because the number of deaths per day did not allow reliable inference.

For the summary presented in this manuscript, we included all countries in the weekly analysis except countries with average weekly coefficient of variation (CV i.e. the ratio of standard deviation to the mean) of the reported deaths between 8th March and 29th November 2020 greater than 1.1 (the 60th quantile of the distribution of CV across all countries). This criterion resulted in the exclusion of 53 countries. 81 countries were included in the final analysis.

We assumed a gamma distributed serial interval with mean 6.48 days and standard deviation of 3.83 days

following [8]. For simplicity, we assumed that the delay in reporting a death is the same as the delay from onset to a case being reported. For the DeCa model, we assumed that the delay in reporting of deaths follows a gamma distribution with mean of 10 days, and standard deviation of 2 days. These figures are roughly consistent with an onset-to-death delay of 15.9 days [9] and onset-to-diagnosis delay of 6.6–6.8 days [10]. The serial interval and delay distributions were discretised using R package EpiEstim [4]. We used a country-specific population-adjusted IFR estimated using the IFR reported in the REACT study (SI Sec. 5).

4.1 Cleaning and pre-processing steps

We used the number of cases and deaths reported by the World Health Organisation (WHO) in the COVID-19 situation report [11]. If either the number of cases or deaths was negative for any country in WHO data, we used the corresponding figures from the data collated by the European Centre for Disease Prevention and Control [12] (if they were non-negative). If both these sources reported negative numbers, we replaced the negative count on a day with the average of the previous and subsequent 3 days. The deaths time series for each country was then visually inspected and any anomalies (e.g. when a large number of deaths were reported on a single day as a correction) were manually corrected using media reports or alternative sources. A complete list of corrections applied to the data is available on the github repository of this project (`https://github.com/mrc-ide/covid19-forecasts-orderly/blob/main/src/prepare_ecdc_data/prepare_ecdc_data.R`).

4.2 Inclusion/Exclusion Criteria

For the analysis carried out every week, we defined a country as having evidence of active transmission if at least 100 deaths had been reported in a country, and at least ten deaths were observed in the country in each of the past two weeks. Forecasts were produced every Monday for the week ahead (Monday to Sunday) using data reported up to the previous day.

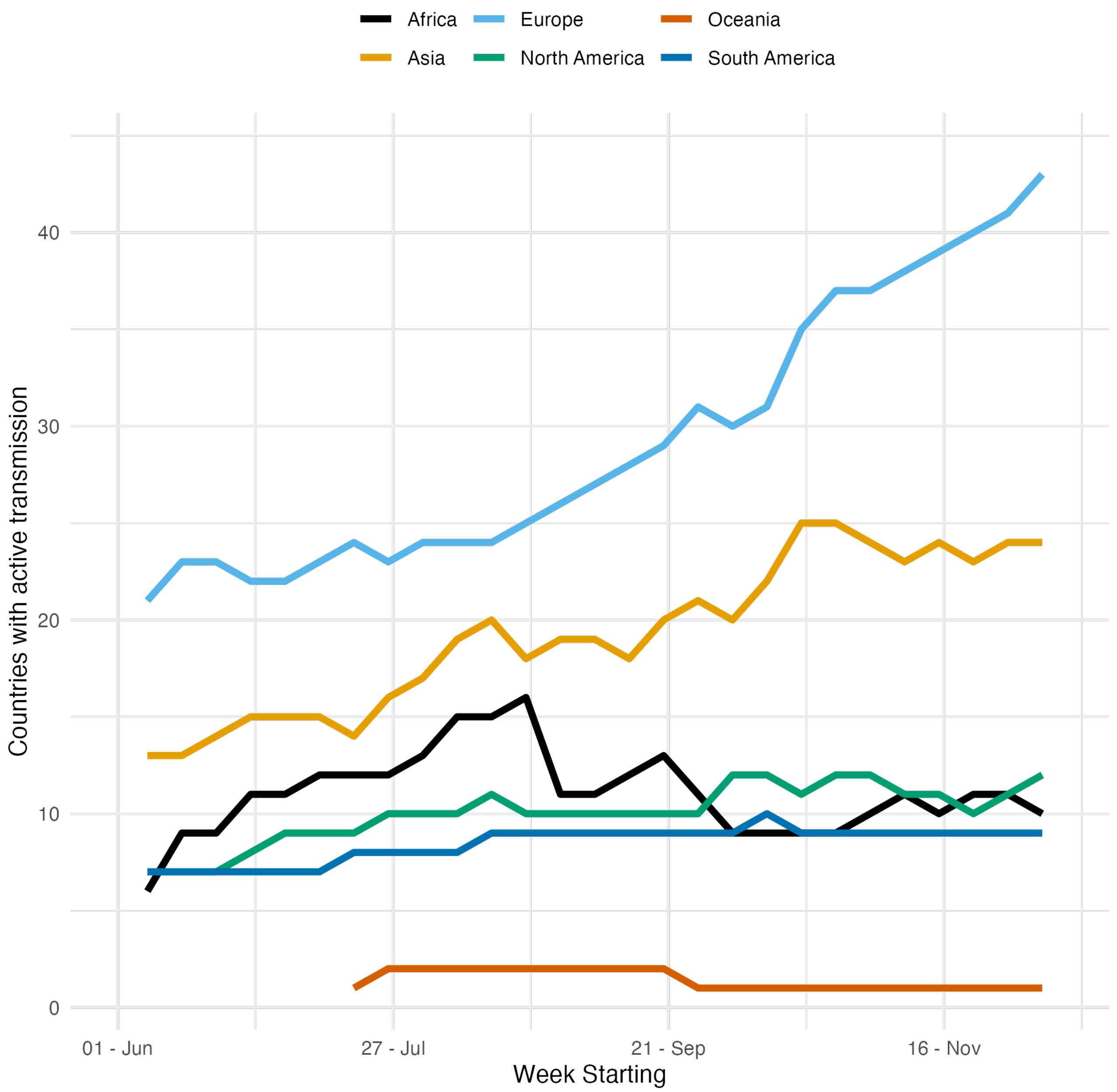

Figure 1. Number of countries included in the weekly reports from 8th March to 29th November 2020. the number of countries included in the weekly analysis grew from 2 in the first week (week starting 8th March 2020), to 94 in the last week of analysis included here (week starting 29th November 2020). Note that some countries that were included in the weekly reports have been excluded from the analysis presented in the manuscript if the average weekly coefficient of variation of the reported deaths between 8th March and 29th November 2020 was greater than 1.1.

5 Infection Fatality Ratio (IFR)

To obtain a IFR distribution, we used the reported deaths and the estimated number of infections in age groups 15-44, 45-64, 65-74 years in the United Kingdom [13]. We first drew 10000 samples from a normal distribution with mean the estimated mean number of infections and standard deviation set to half the width of the 95% CI divided by 1.96. We divided the reported number of deaths in the corresponding age groups by the estimated number of infections to obtain age-disaggregated IFR distributions. We then obtained a country-specific IFR distribution as a weighted sum of the age-disaggregated IFR where the weights are the proportion of the total population in each group in a country [14].

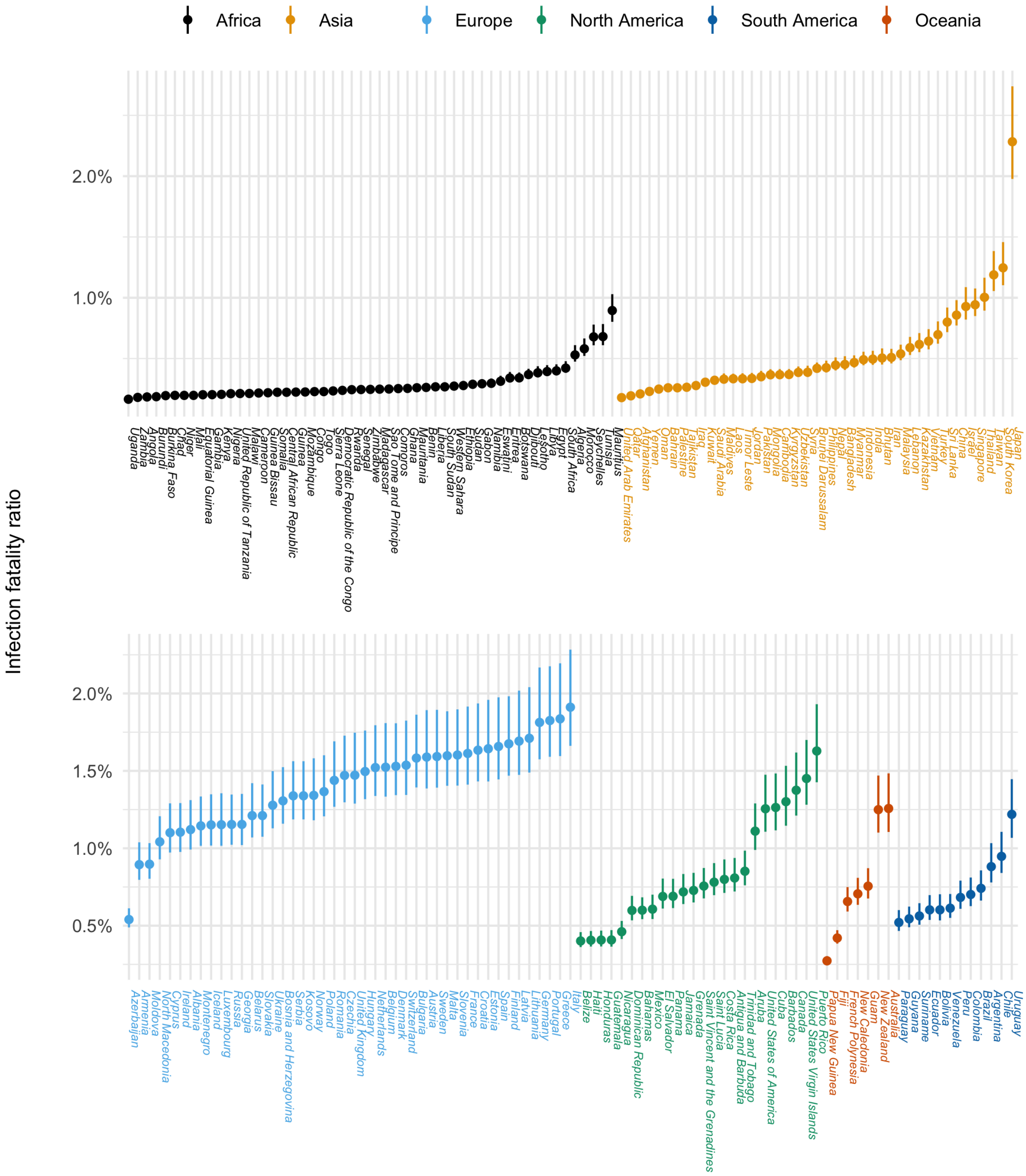

Figure 2. Population adjusted IFR distribution. The solid dots indicate the median estimate and the vertical bars represent the 95% CrI.

6 Augmentation of observed cases for DeCa

In the DeCa model, forecasts of deaths at time t rely on the number of cases from the beginning of the time series to time t. We obtained a distribution of cases in the week for which we are producing forecasts by sampling from a gamma distribution with the mean and standard deviation equal to those of the most recent week of data on cases. We illustrate this process and also show that this does not influence the results under the chosen distribution of delays from case report to death (SI Fig. 3).

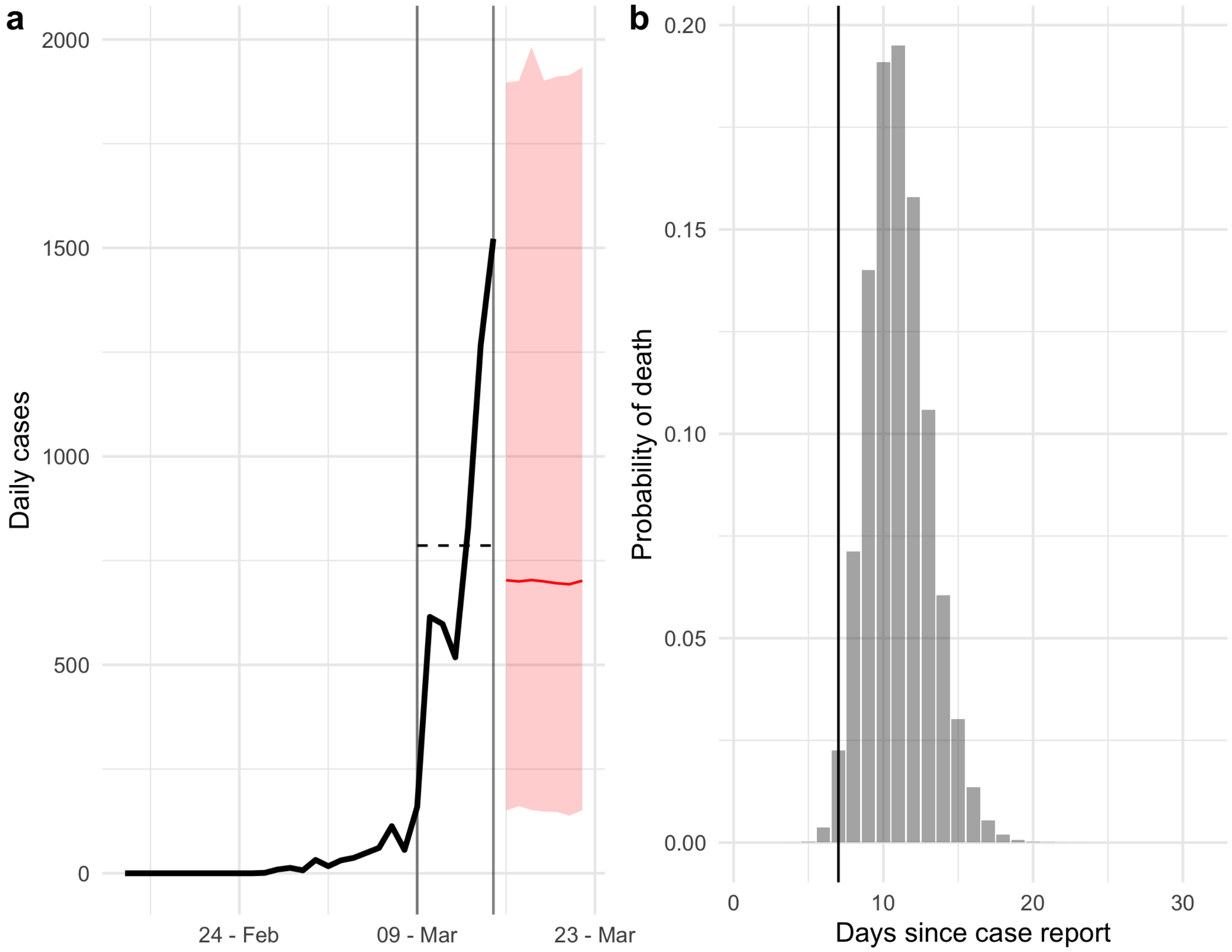

Figure 3. (a) The observed time series of cases (thick black line) is augmented by sampling from a gamma distribution with the mean and standard deviation of the cases in the most recent week of data. The vertical lines indicate the last week and the dashed horizontal line is the mean of the cases in this period. The red line and the shaded area represent the median and the 95% CrI of the sampled cases. (b) The probability distribution of delays from case report to death. For a case reported at time t, the probability of death within d days is the sum of probabilities from 0 to d. In particular, the probability that a case will die within a week (our short-term forecast horizon) is the sum of probabilities to the left of the vertical line (7 days), which is approximately 2%.

7 Model performance assessment

7.1 Mean relative error by epidemic phase

Epidemic phase	Proportion in 50% CrI	Proportion in 95% CrI	MRE
Likely decreasing	63.9% (29.7%)	92.1% (17.3%)	0.5 (0.9)
Definitely decreasing	43.2% (32.4%)	79.9% (28.2%)	0.5 (0.8)
Likely growing	60.6% (32.5%)	91.2% (20.0%)	0.4 (0.4)
Definitely growing	50.8% (31.8%)	85.4% (24.8%)	0.3 (0.2)
Likely stable	52.7% (31.7%)	84.0% (25.0%)	0.3 (0.2)
Indeterminate	70.8% (29.1%)	96.3% (12.5%)	0.5 (0.5)

Table 1. Coverage probability and mean relative error of short-term forecasts in each epidemic phase defined retrospectively using R_T^{curr}. For each metric, we show the mean and standard deviation (in parentheses) across countries and weeks of forecast.

7.2 Relative error and comparison with no-change model

This section presents the mean relative error of the model and comparison of the model error with the error made by a model that uses the average of the past 10 days as the forecast for the week ahead.

(a)

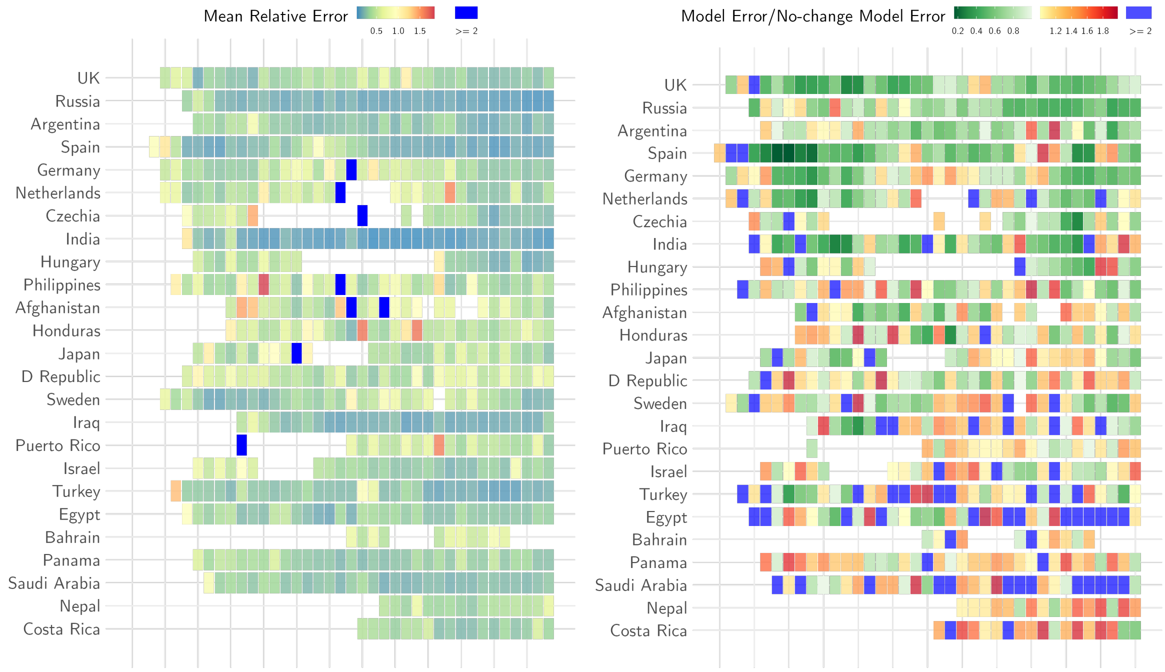

(b)

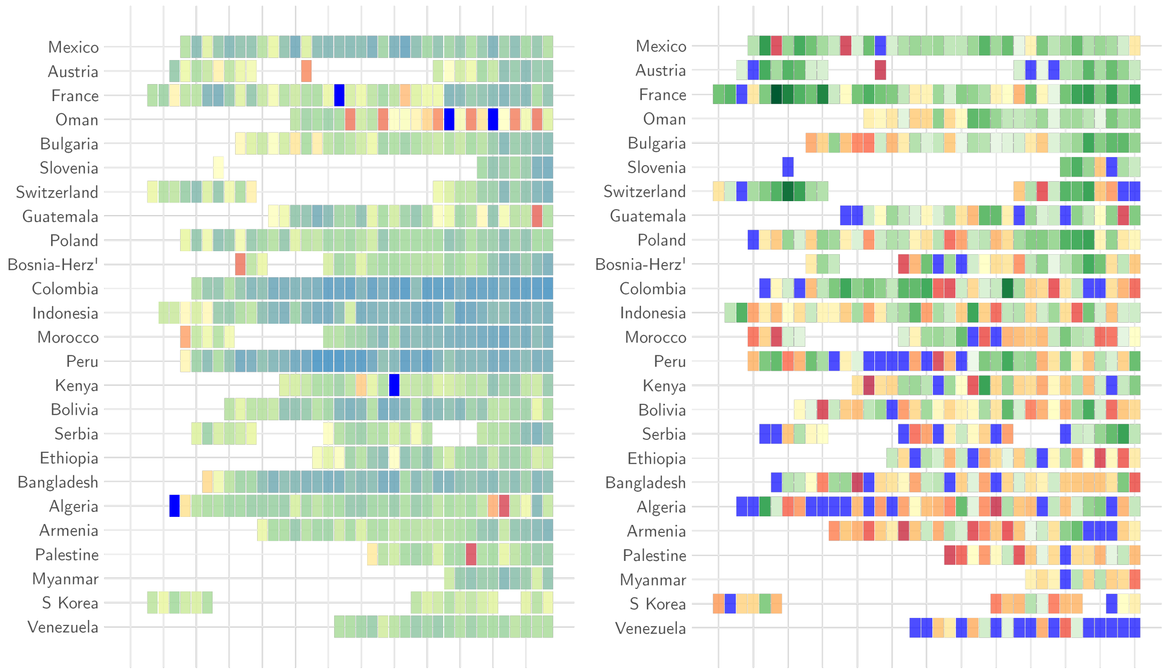

(c)

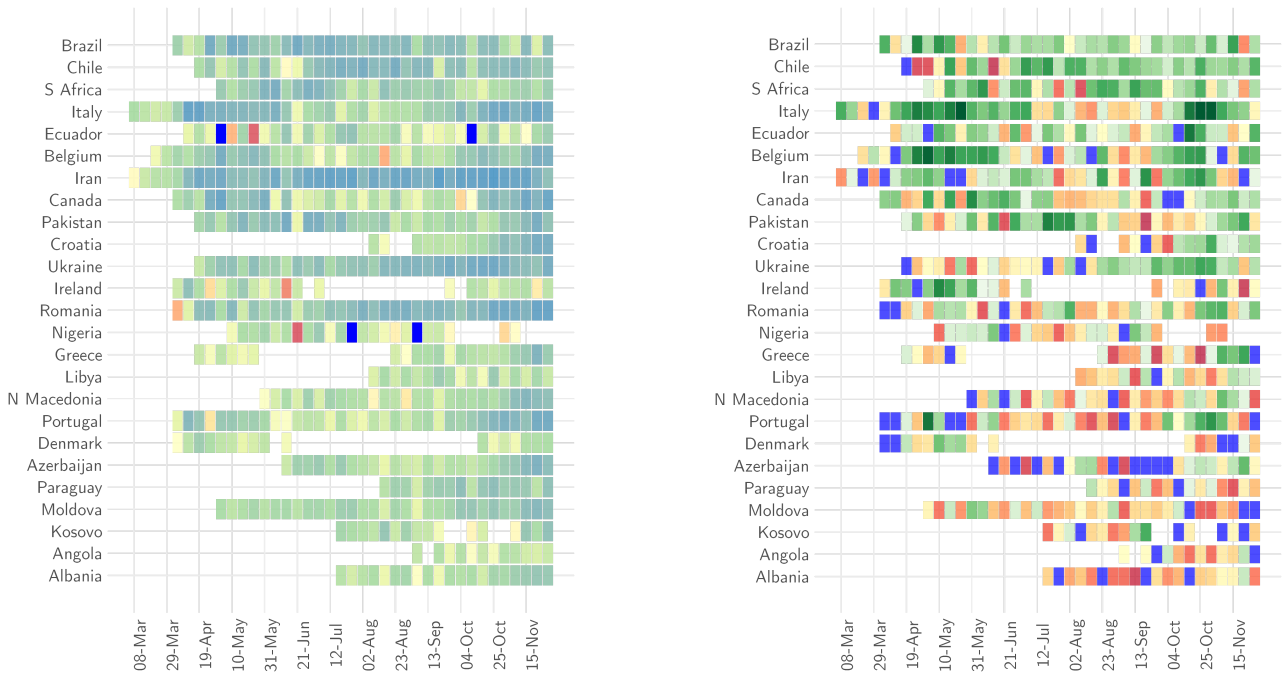

Figure 4. Mean relative error and comparison with null model In each panel, the left graph shows the relative error of the ensemble model for each week of forecast (x-axis) and for each country (y-axis). Dark blue cells indicate weeks where the relative error of the model was greater than 2. The right panel shows the ratio of the absolute error of the model to the absolute error of the no-change null model. Shades of green show weeks for a given country where the ratio was smaller than 1 i.e., the model error was smaller, and weeks where the ratio was greater than 1 i.e. the model error was bigger than the null model error are shown in shades of red (yellow to red). Dark blue cells indicate weeks where the ratio was greater than 2. Panels (a) - (c) show results for all countries included in the analysis. Bosnia-Herz: Bosnia and Herzegovina, D Republic: Dominican Republic, N Macedonia: North Macedonia, S Africa: South Africa, S Korea: South Korea, USA: United States of America, UK: United Kingdom.

7.2.1 Comparison with no-change and linear models by phase

Phase	Ensemble model error <no-change model error	Ensemble model error <Linear model error	Weeks
Likely decreasing	42.7% (128)	78.7% (236)	300
Definitely decreasing	76.8% (192)	90.8% (227)	250
Likely growing	30.7% (115)	75.5% (283)	375
Definitely growing	74.4% (364)	79.6% (389)	489
Likely stable	39.1% (79)	82.2% (166)	202
Indeterminate	24.7% (116)	77.7% (365)	470

Table 2. Comparison of the absolute error of the ensemble model with that made by a null no-change model or a predictions from a linear model as forecast for the week ahead for each phase of the pandemic defined retrospectively using R_T^{curr}. The right-most column (Weeks) shows the total number of weeks in a given phase.

7.3 Relative error and comparison with a linear model

This section presents the relative error of the ensemble model and comparison of the model error with the error of a linear model (a line fitted to the past 10 observations). The linear model was fitted in rstannarm [15] and the forecasts were sampled from the posterior predictive distribution.

(a)

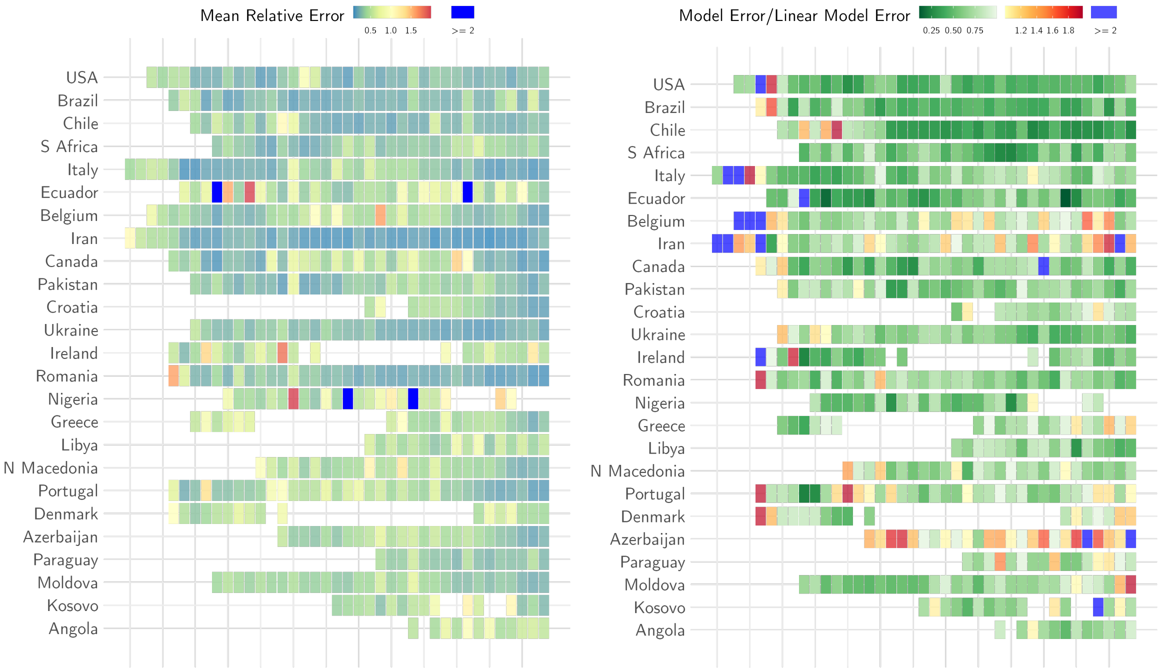

Mean Relative Error
0.5 1.0 1.5
>= 2
Model Error/Linear Model Error
0.25 0.50 0.75
1.2 1.4 1.6 1.8
>= 2
USA
Brazil
Chile
S Africa
Italy
Ecuador
Belgium
Iran
Canada
Pakistan
Croatia
Ukraine
Ireland
Romania
Nigeria
Greece
Libya
N Macedonia
Portugal
Denmark
Azerbaijan
Paraguay
Moldova
Kosovo
Angola

(b)

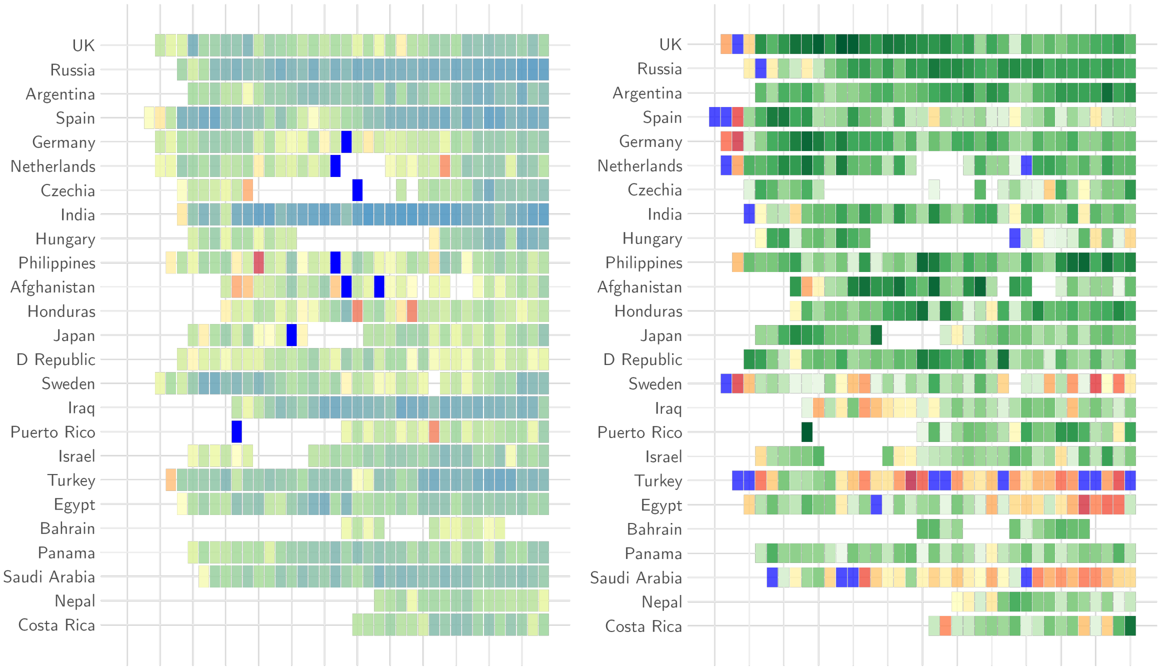

UK
Russia
Argentina
Spain
Germany
Netherlands
Czechia
India
Hungary
Philippines
Afghanistan
Honduras
Japan
D Republic
Sweden
Iraq
Puerto Rico
Israel
Turkey
Egypt
Bahrain
Panama
Saudi Arabia
Nepal
Costa Rica

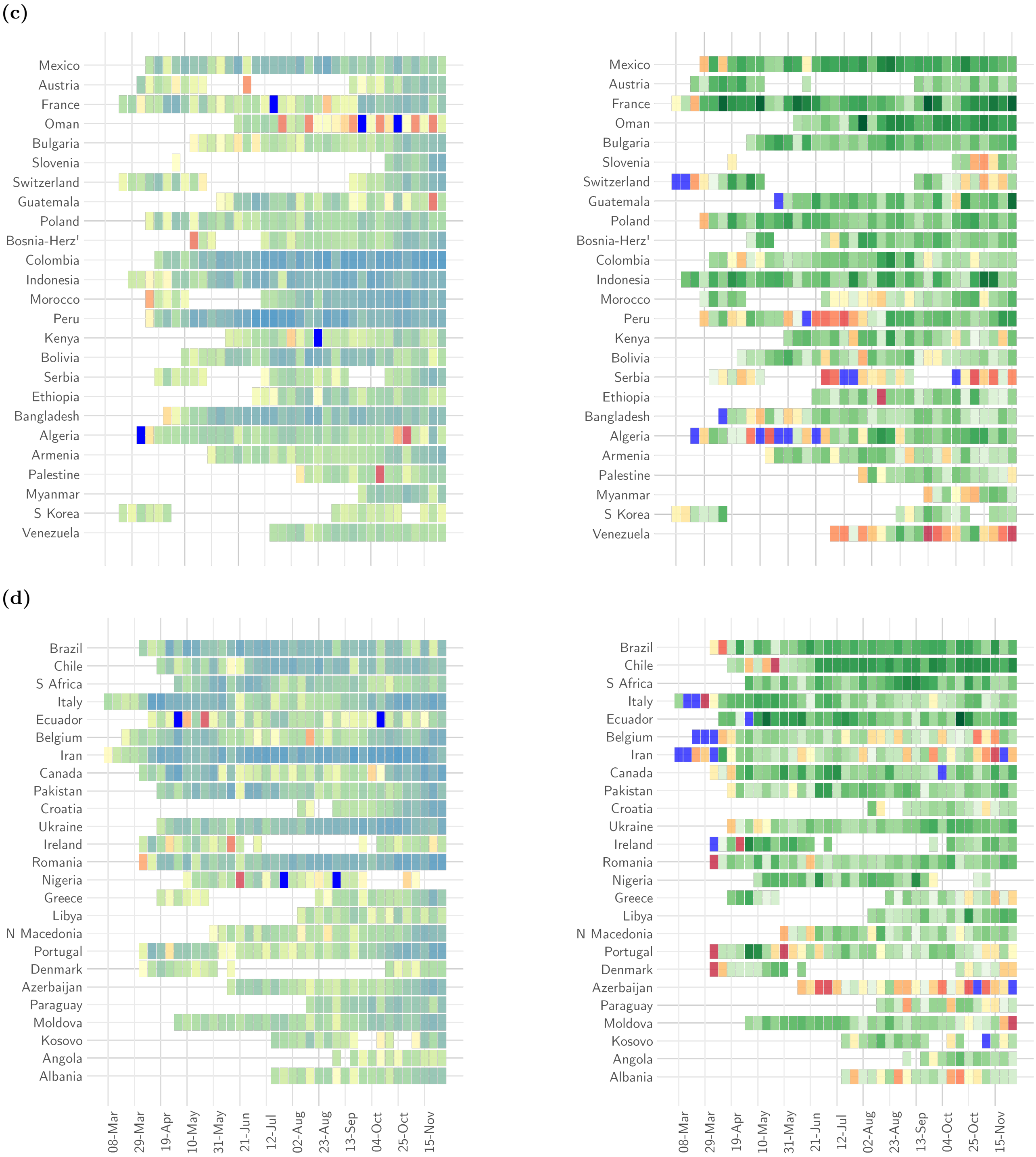

Figure 5. Relative error and comparison with a linear model In each panel, the left graph shows the mean relative error of the model for each week of forecast (x-axis) and for each country (y-axis). Dark blue cells indicate weeks where the relative error of the model was greater than 2. The right panel shows the ratio of the absolute error of the model to the absolute error of forecasts made using a linear model. Shades of green show weeks for a given country where the ratio was smaller than 1 i.e., the model error was smaller, and weeks where the ratio was greater than 1 i.e. the model error was bigger than the null model error are shown in shades of red (yellow to red). Dark blue cells indicate weeks where the ratio was bigger than 2. Panels (a)-(d) show results for all countries included in the analysis. Bosnia-Herz: Bosnia and Herzegovina, D Republic: Dominican Republic, N Macedonia: North Macedonia, S Africa: South Africa, S Korea: South Korea, USA: United States of America, UK: United Kingdom.

7.4 Mean relative error compared with the weekly CV

The relative error of the model was proportional to the CV of the number of deaths reported each week and inversely proportional to the weekly incidence.

(a) **(b)**

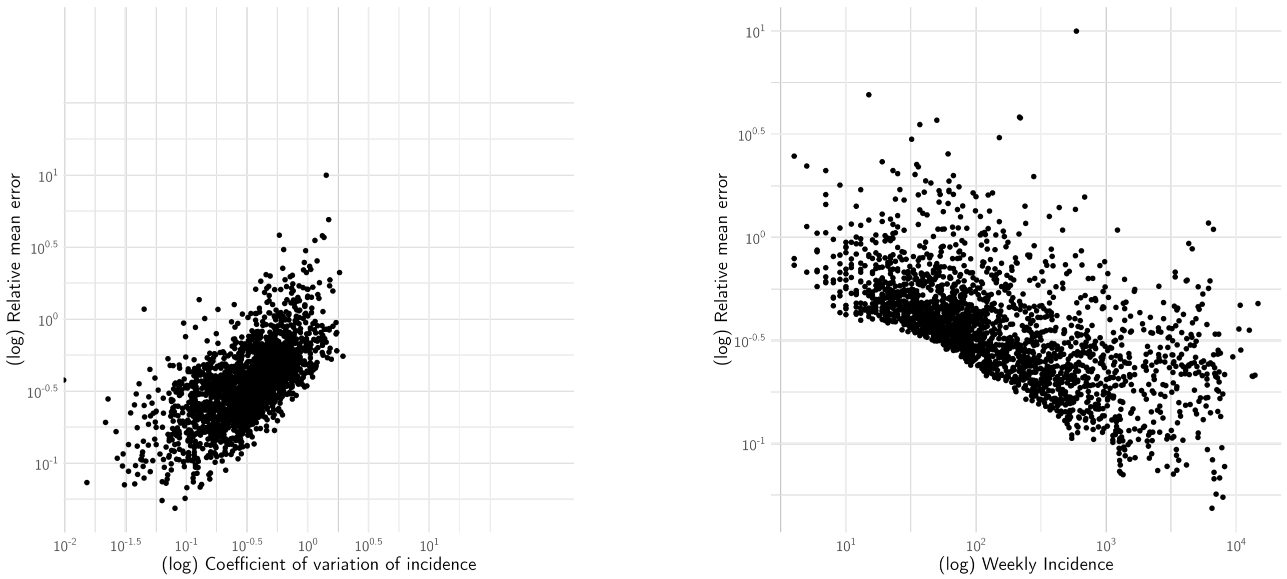

Figure 6. The log MRE scales linearly with the log weekly CV (a) and inversely with the log weekly incidence (b).

7.5 Coverage Probability

This section presents the proportion of observations in 50% CrI and 95% CrI for each country and each week of forecast.

Proportion of observations in 50% CrI

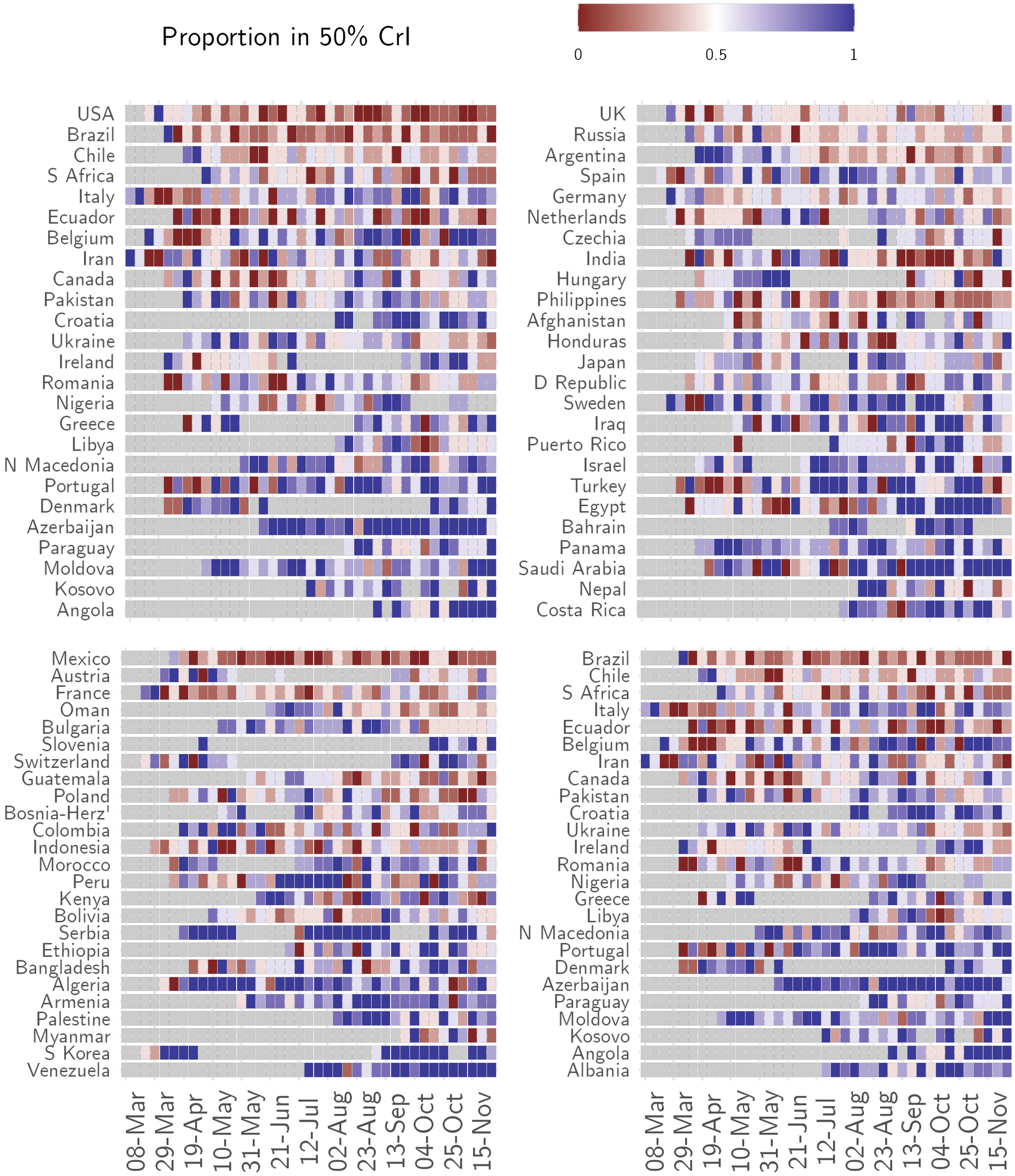

Figure 7. For each week of forecast (x-axis) and each country (y-axis), the proportion of observations in the 50% CrI of the forecasts. Gray cells indicate weeks where a country was not included in the analysis because the number of deaths did not meet the threshold (see SI Sec. 4).

Proportion of observations in 95% CrI 198

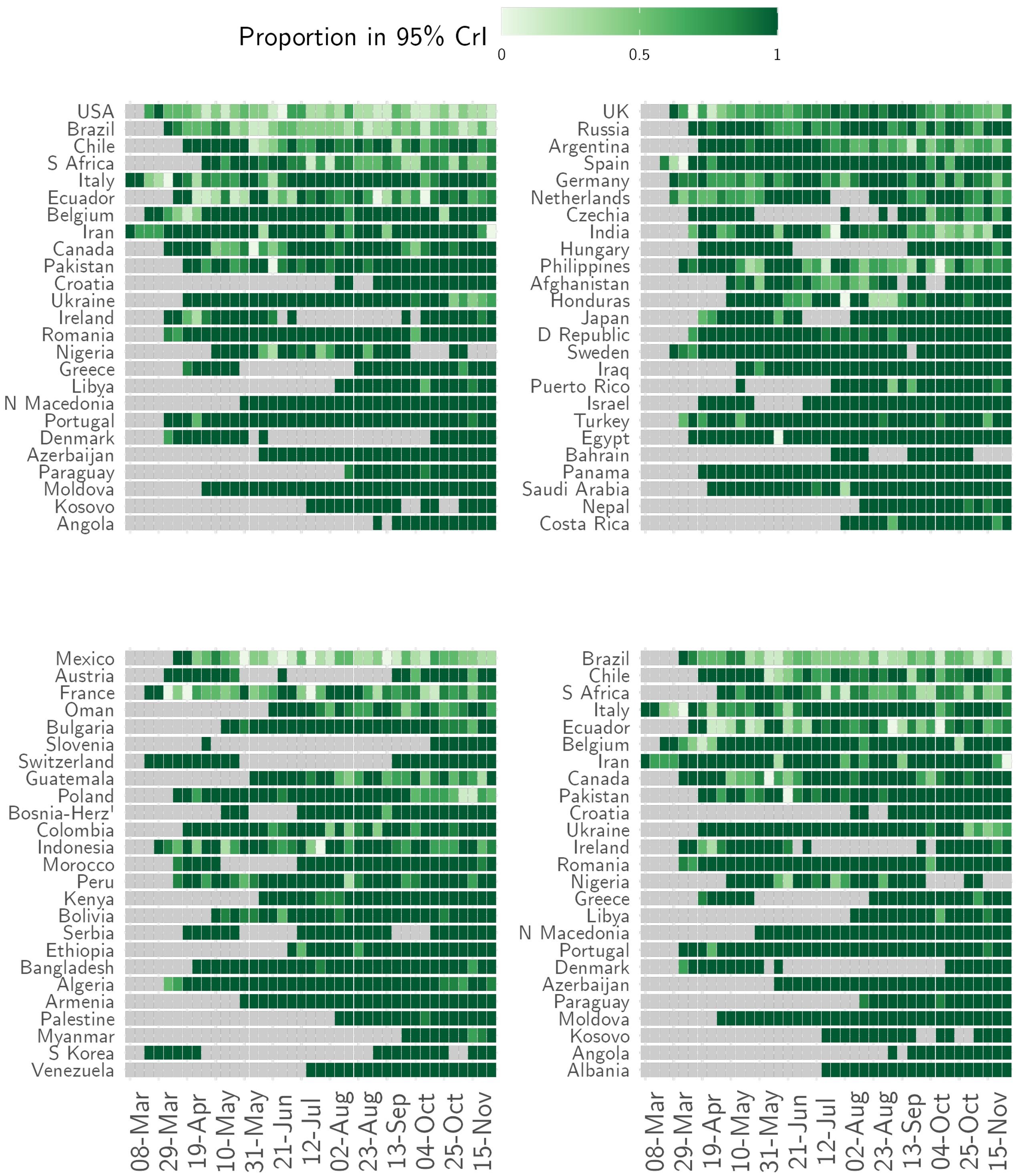

Figure 8. For each week of forecast (x-axis) and each country (y-axis), the proportion of observations in 95% CrI of the forecasts. Gray cells indicate weeks where a country was not included in the analysis because the number of deaths did not meet the threshold (see SI Sec. 4). Bosnia-Herz: Bosnia and Herzegovina, D Republic: Dominican Republic, N Macedonia: North Macedonia, S Africa: South Africa, S Korea: South Korea, USA: United States of America, UK: United Kingdom.

8 Medium-term forecasts

This section presents the performance assessment results for medium-term forecasts. The relative error for each country and week of forecast are presented in (SI Sec. 8.1) and coverage probability are shown in (SI Sec. 8.2).

8.1 Relative error

202

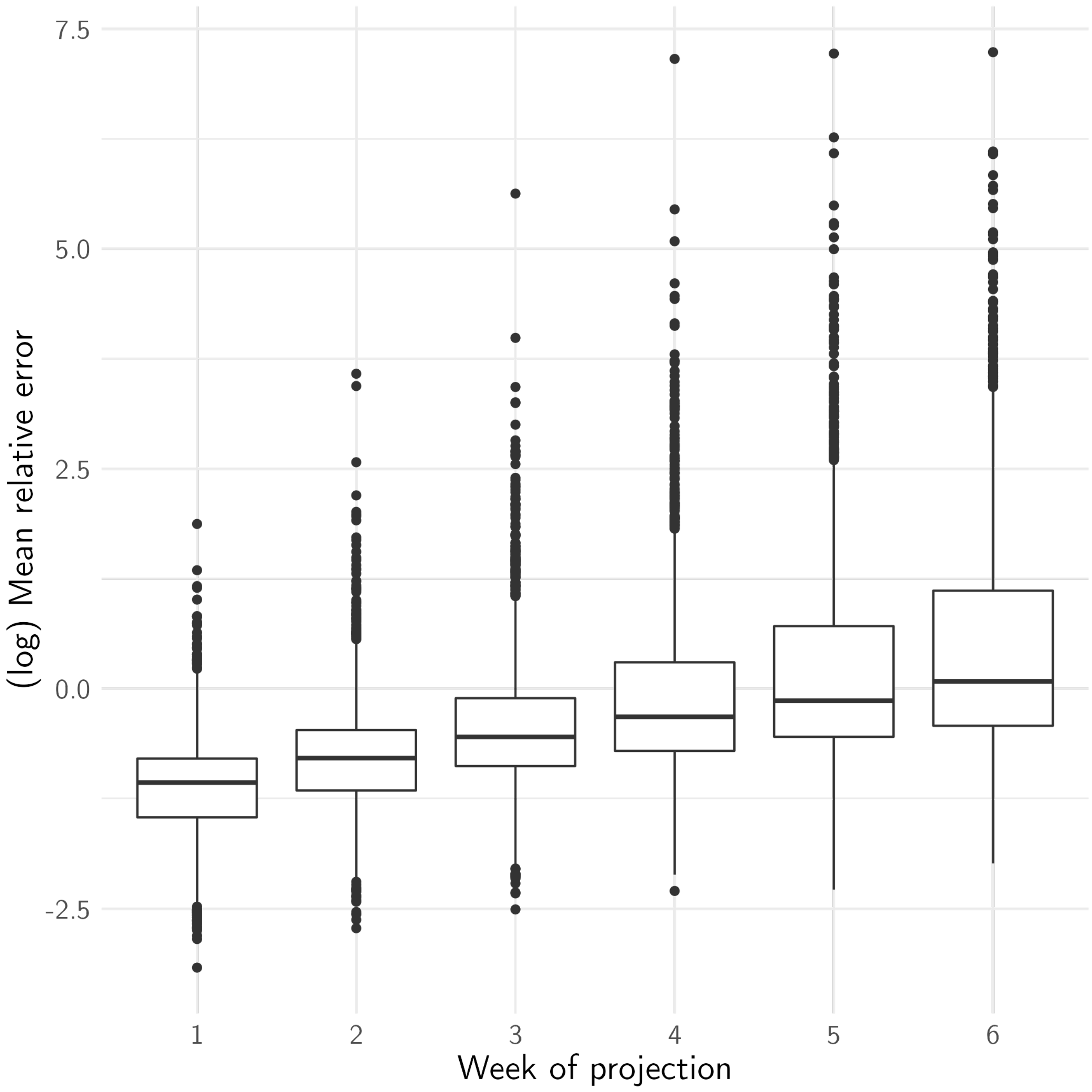

Figure 9. The MRE grew over the projection horizon becoming unacceptably high beyond a 4-week horizon. Note that the MRE is presented on a logarithmic scale. That is, 0 represents an MRE of 1; a perfect model with MRE 0 would be indicated here by negative infinity.

(a)

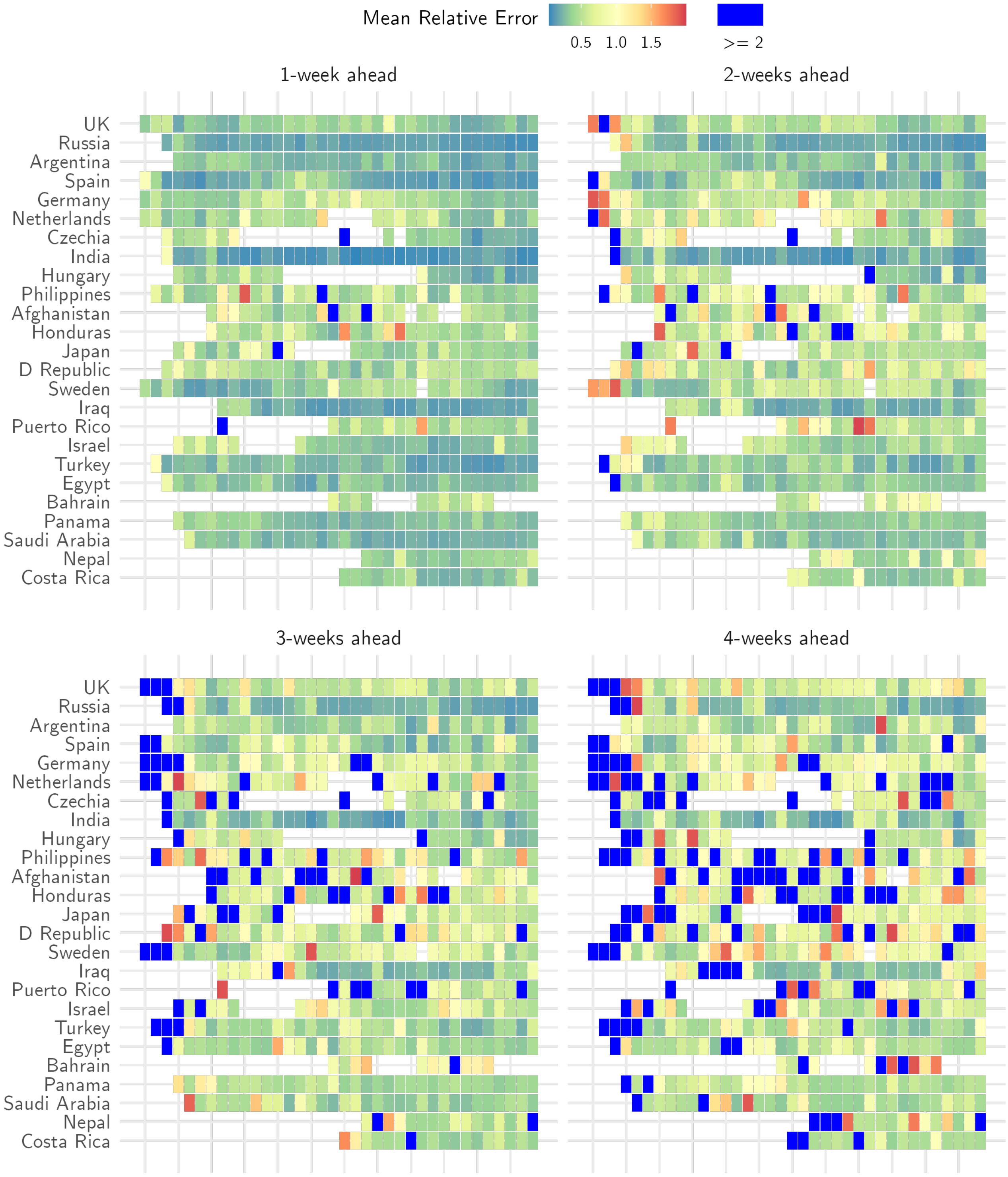
Mean Relative Error
0.5
1.0
1.5
>= 2
1-week ahead
2-weeks ahead
3-weeks ahead
4-weeks ahead
UK
Russia
Argentina
Spain
Germany
Netherlands
Czechia
India
Hungary
Philippines
Afghanistan
Honduras
Japan
D Republic
Sweden
Iraq
Puerto Rico
Israel
Turkey
Egypt
Bahrain
Panama
Saudi Arabia
Nepal
Costa Rica

(b)

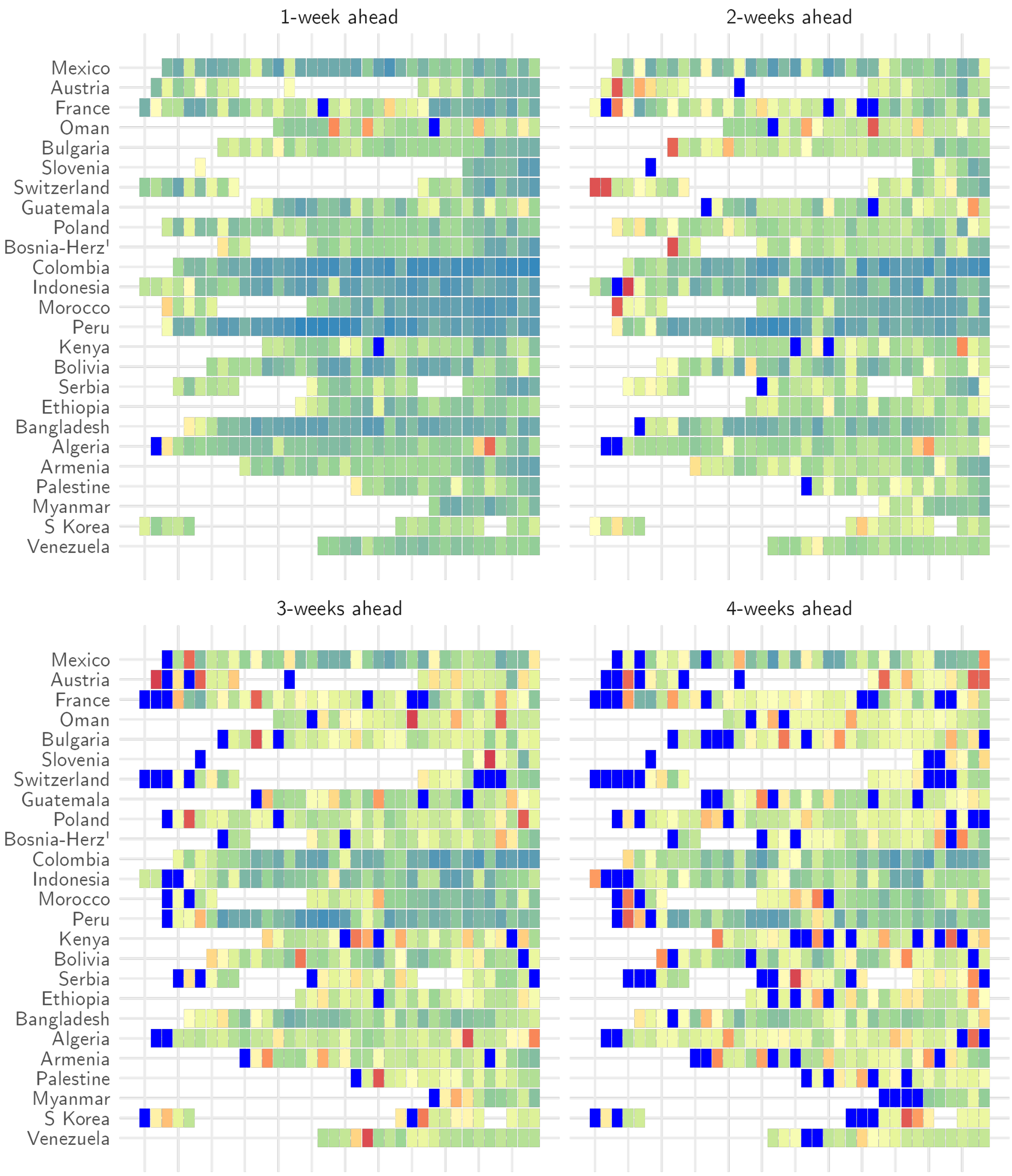
1-week ahead
2-weeks ahead
3-weeks ahead
4-weeks ahead
Mexico
Austria
France
Oman
Bulgaria
Slovenia
Switzerland
Guatemala
Poland
Bosnia-Herz'
Colombia
Indonesia
Morocco
Peru
Kenya
Bolivia
Serbia
Ethiopia
Bangladesh
Algeria
Armenia
Palestine
Myanmar
S Korea
Venezuela

(c)

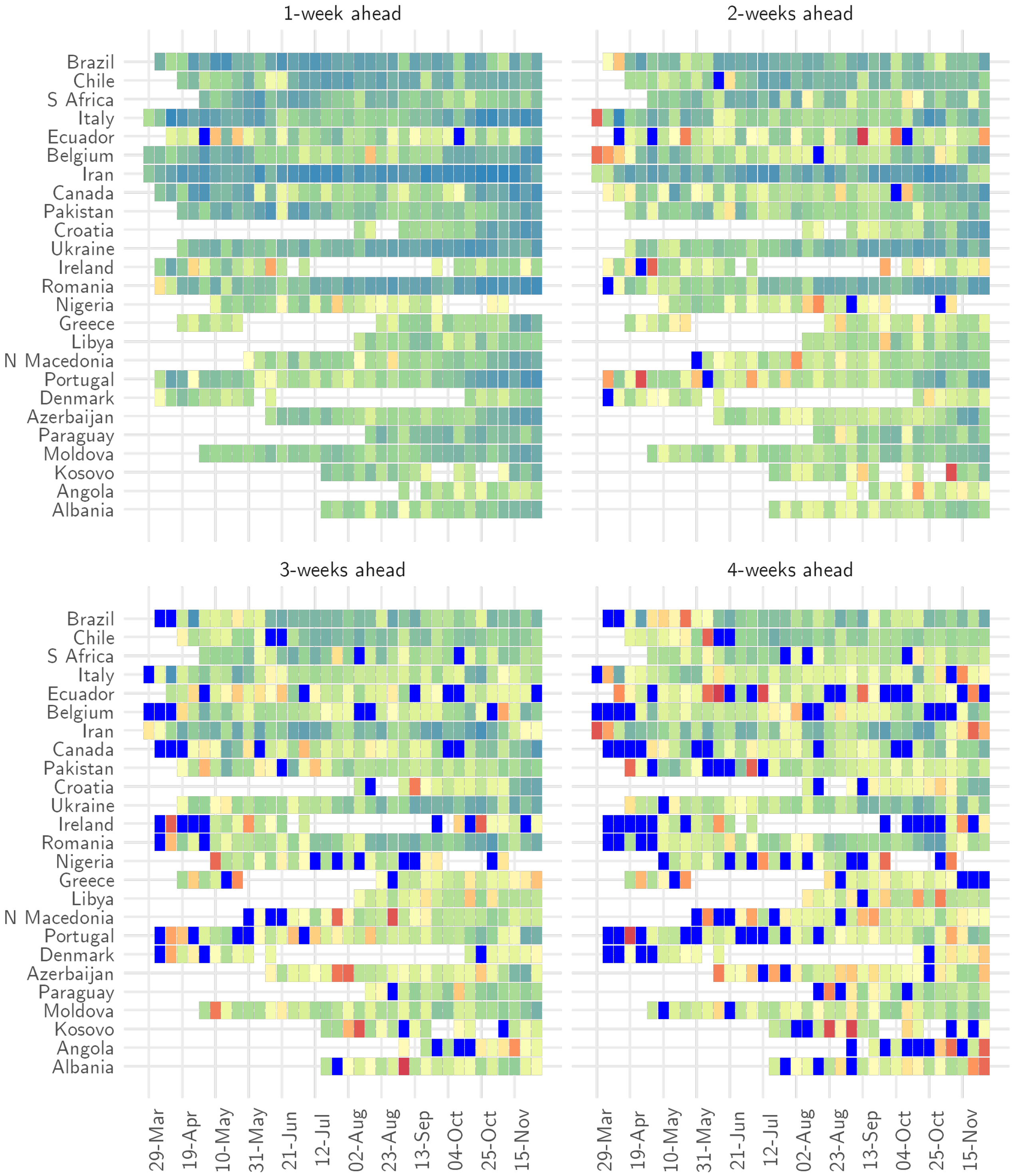

Figure 10. Mean relative error of medium-term forecasts. The relative error of the model in 1-week, 2-week , 3-week, and 4-week ahead forecasts for each week of forecast (x-axis) and for each country (y-axis). Dark blue cells indicate weeks where the relative error of the model was greater than 2. Panels (a)-(c) present results for all countries included in the analysis. Bosnia-Herz: Bosnia and Herzegovina, D Republic: Dominican Republic, N Macedonia: North Macedonia, S Africa: South Africa, S Korea: South Korea, USA: United States of America, UK: United Kingdom.

Week of forecast	MRE <0.5	MRE <1
1	80.8%	91.1%
2	58.3%	89.5 %
3	33.2%	78.3%
4	25.6%	66.0%

Table 3. The MRE of medium-term forecasts remained relatively small over a 4-week forecast horizon. The MRE was less than 1 in 66.0% and less than 0.5 in 25.6% of weeks in 4-week ahead forecasts.

8.2 Coverage Probability

Proportion of observations in 50% CrI

(a)

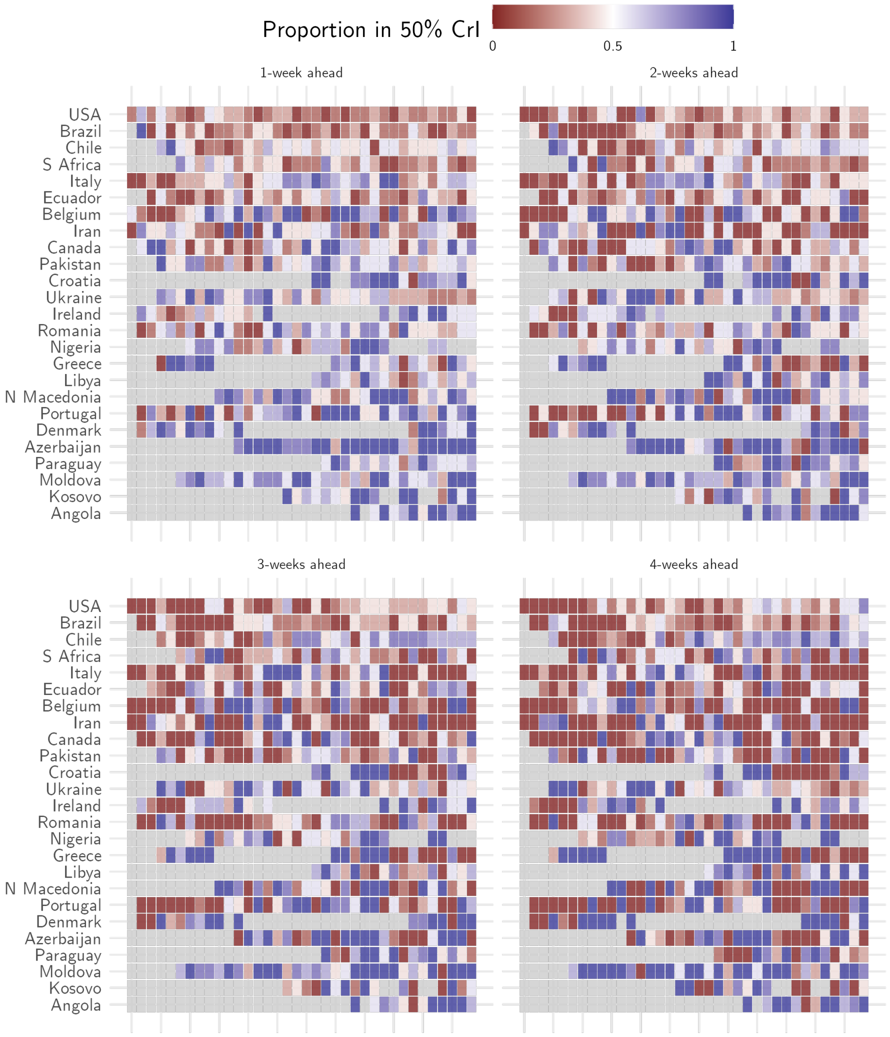

(b)

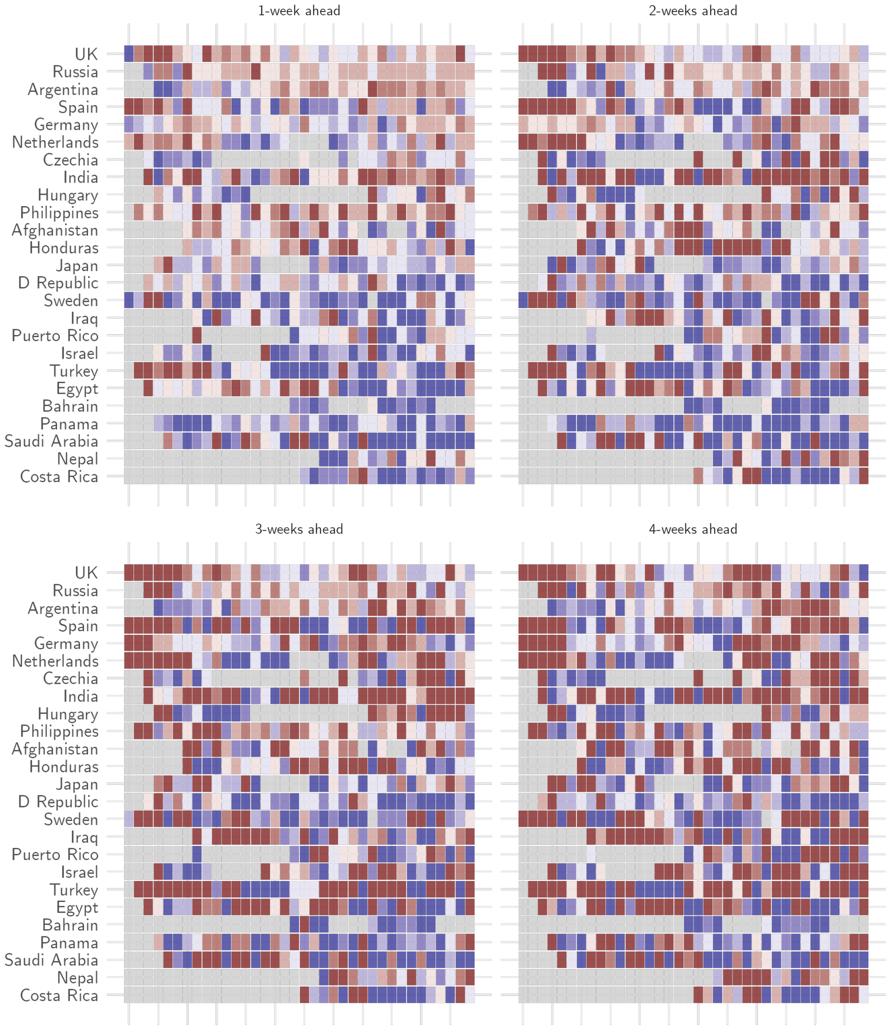

1-week ahead
2-weeks ahead
3-weeks ahead
4-weeks ahead
UK
Russia
Argentina
Spain
Germany
Netherlands
Czechia
India
Hungary
Philippines
Afghanistan
Honduras
Japan
D Republic
Sweden
Iraq
Puerto Rico
Israel
Turkey
Egypt
Bahrain
Panama
Saudi Arabia
Nepal
Costa Rica

(c)

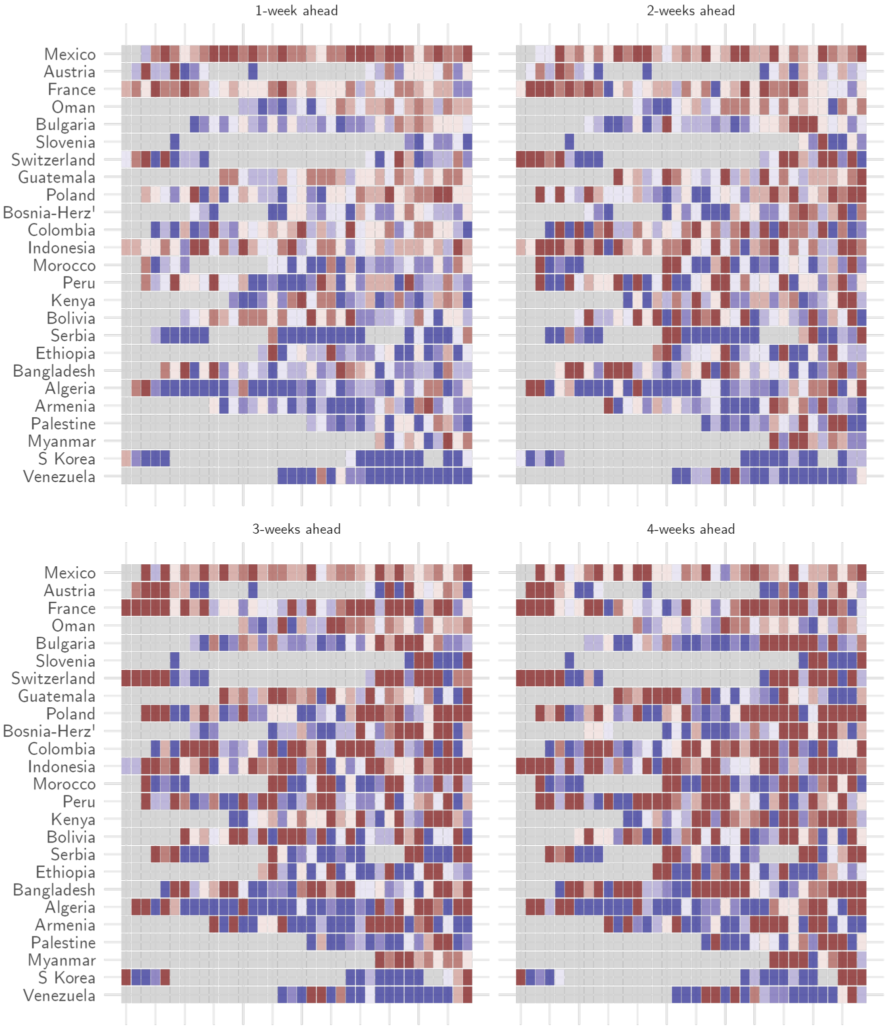
1-week ahead
2-weeks ahead
3-weeks ahead
4-weeks ahead
Mexico
Austria
France
Oman
Bulgaria
Slovenia
Switzerland
Guatemala
Poland
Bosnia-Herz'
Colombia
Indonesia
Morocco
Peru
Kenya
Bolivia
Serbia
Ethiopia
Bangladesh
Algeria
Armenia
Palestine
Myanmar
S Korea
Venezuela

(d)

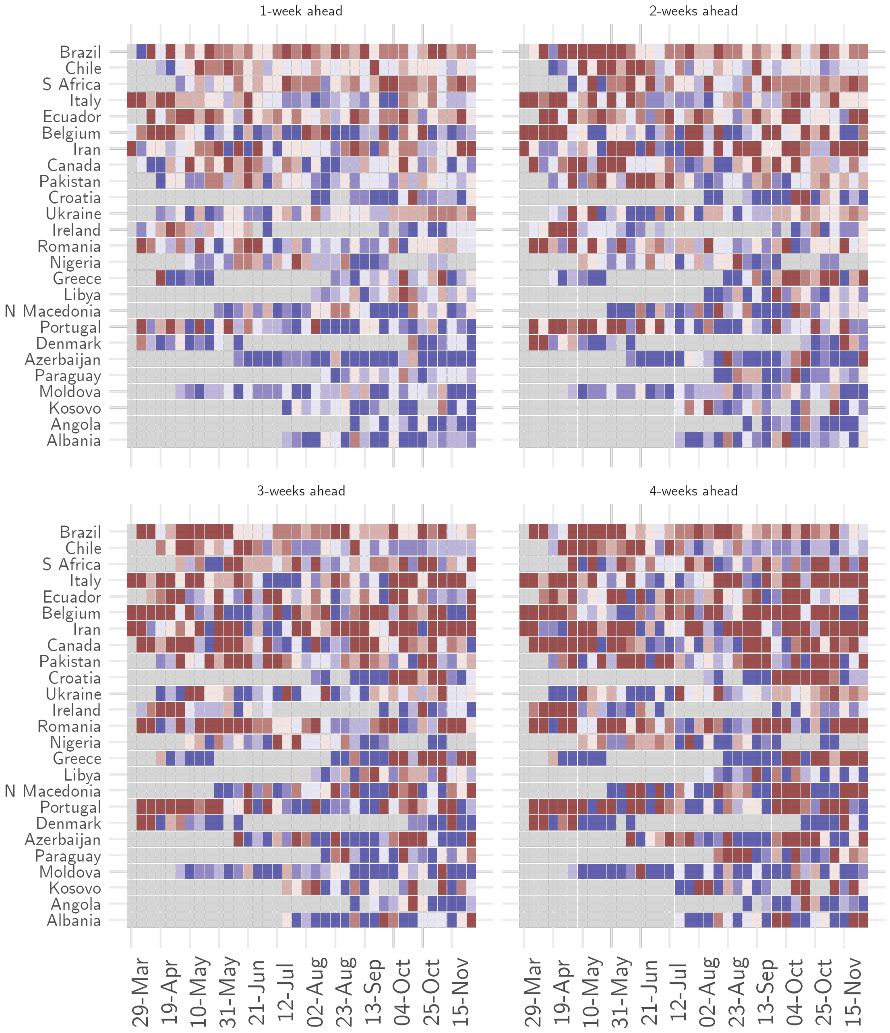

Figure 11. The proportion of observations in the 50% CrI of the forecasts for 1-week, 2-week , 3-week, and 4-week (clockwise from top left) ahead for each week of forecast (x-axis) and for each country (y-axis). Panels (a)-(d) present results for all countries included in the analysis. Gray cells indicate weeks where a country was not included in the analysis because the number of deaths did not meet the threshold (see SI Sec. 4). Bosnia-Herz: Bosnia and Herzegovina, D Republic: Dominican Republic, N Macedonia: North Macedonia, S Africa: South Africa, S Korea: South Korea, USA: United States of America, UK: United Kingdom.

Proportion of observations in 95% CrI

(a)

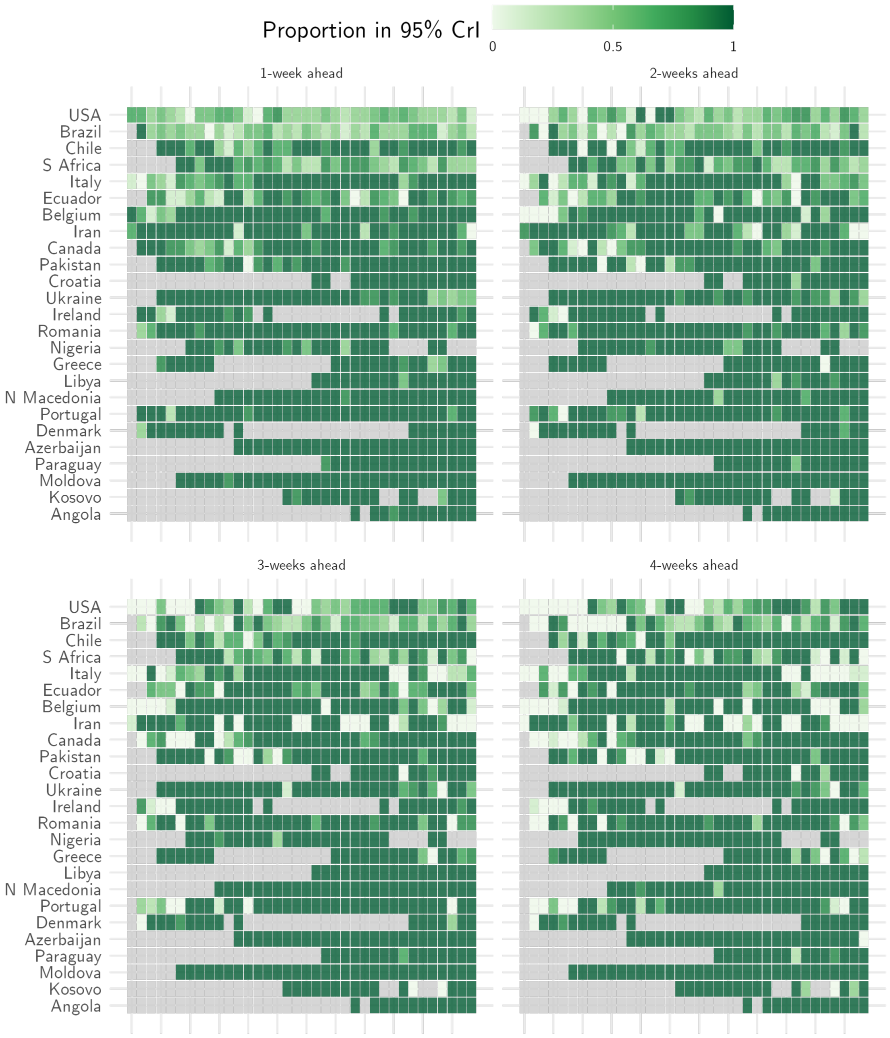

(b)

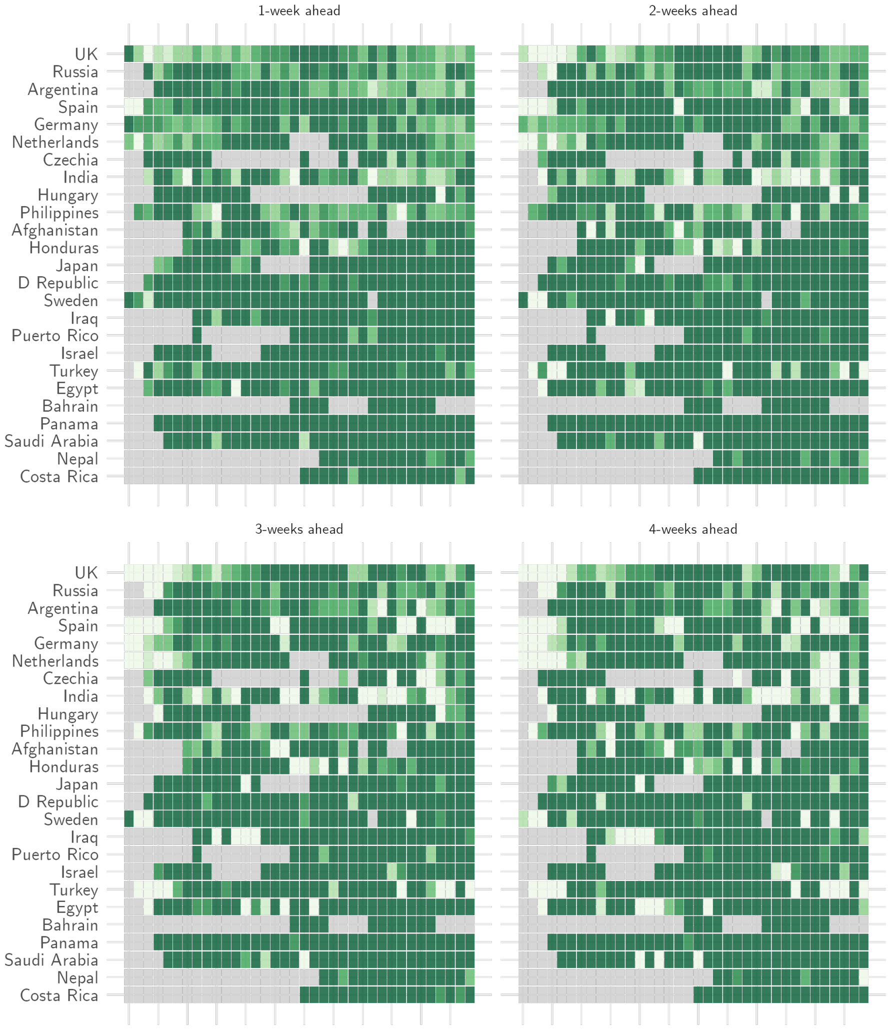
1-week ahead
2-weeks ahead
3-weeks ahead
4-weeks ahead
UK
Russia
Argentina
Spain
Germany
Netherlands
Czechia
India
Hungary
Philippines
Afghanistan
Honduras
Japan
D Republic
Sweden
Iraq
Puerto Rico
Israel
Turkey
Egypt
Bahrain
Panama
Saudi Arabia
Nepal
Costa Rica

(c)

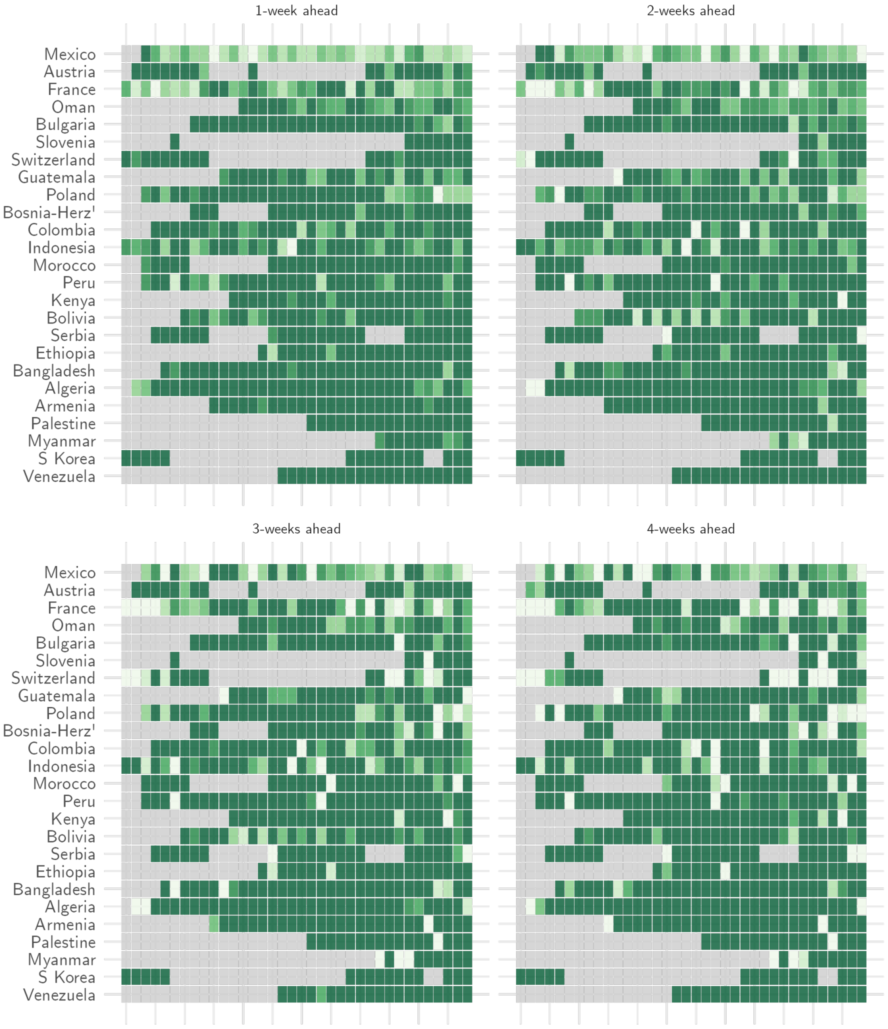
1-week ahead
2-weeks ahead
3-weeks ahead
4-weeks ahead
Mexico
Austria
France
Oman
Bulgaria
Slovenia
Switzerland
Guatemala
Poland
Bosnia-Herz'
Colombia
Indonesia
Morocco
Peru
Kenya
Bolivia
Serbia
Ethiopia
Bangladesh
Algeria
Armenia
Palestine
Myanmar
S Korea
Venezuela

(d)

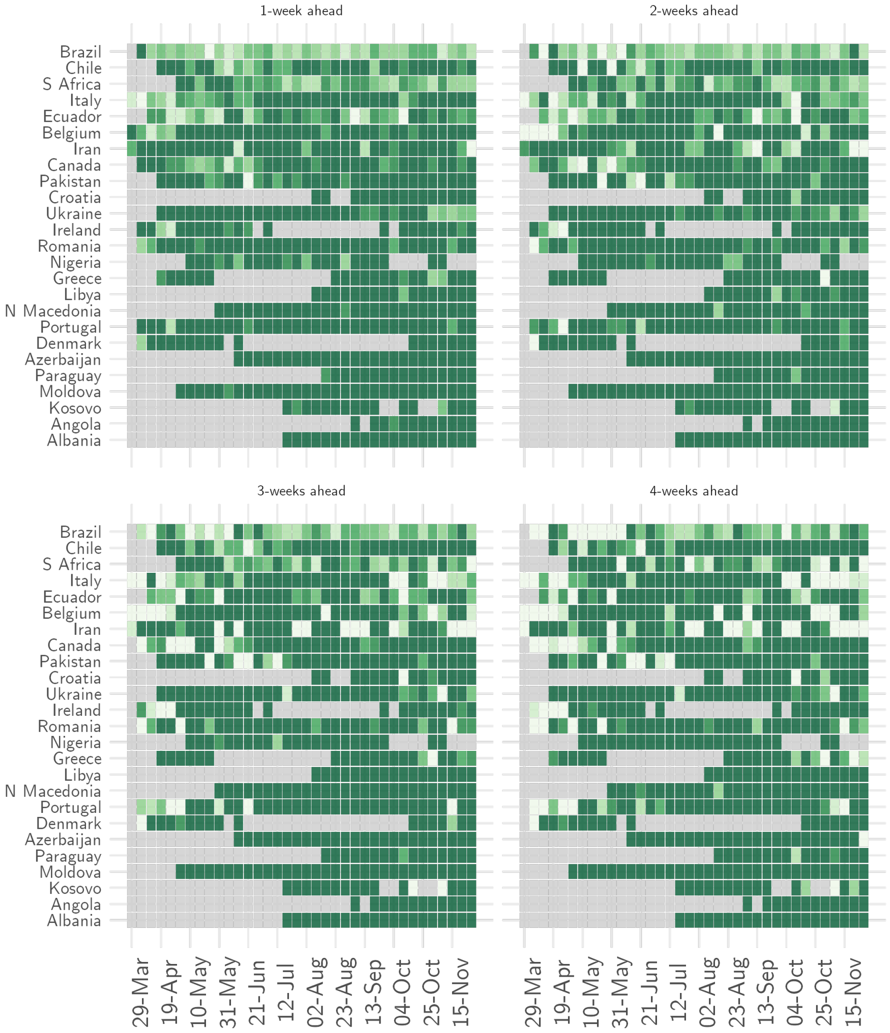

Figure 12. The proportion of observations in the 95% CrI of the forecasts for 1-week, 2-week, 3-week, and 4-week ahead for each week of forecast (x-axis) and for each country (y-axis). Panels (a)-(d) present results for all countries included in the analysis. Gray cells indicate weeks where a country was not included in the analysis because the number of deaths did not meet the threshold (see SI Sec. 4). Bosnia-Herz: Bosnia and Herzegovina, D Republic: Dominican Republic, N Macedonia: North Macedonia, S Africa: South Africa, S Korea: South Korea, USA: United States of America, UK: United Kingdom.

Medium-term phase

Misclassified epidemic phase

Phase using R^{curr}	Phase using R^S				
	Definitely decreasing	Likely decreasing	Definitely growing	Likely growing	Indeterminate
Definitely decreasing	0.00% (0)	100.00% (253)	0.00% (0)	0.00% (0)	0.00% (0)
Likely decreasing	72.73% (328)	0.00% (0)	0.00% (0)	0.00% (0)	27.27% (123)
Definitely growing	0.00% (0)	0.00% (0)	0.00% (0)	56.29% (1513)	43.71% (1175)
Likely growing	0.00% (0)	0.00% (0)	0.92% (30)	0.00% (0)	99.08% (3239)
Indeterminate	1.68% (31)	79.35% (1460)	0.00% (0)	18.97% (349)	0.00% (0)

Table 4. In country-days where the phase definitions using R_t^{curr} (shown along rows) and R_t^S (shown along columns) were different, R_t^S most frequently mis-classified the phase as a phase with greater uncertainty. The numbers in parenthesis indicate the number of country-days for a given combination of phase in row and column.

References

[1] Svensson Å. A note on generation times in epidemic models. Mathematical biosciences. 2007;208(1):300–311.

[2] Nouvellet P, Cori A, Garske T, Blake IM, Dorigatti I, Hinsley W, et al. A simple approach to measure transmissibility and forecast incidence. Epidemics. 2018;22:29–35. doi:10.1016/j.epidem.2017.02.012.

[3] Fraser C. Estimating individual and household reproduction numbers in an emerging epidemic. PloS One. 2007;2(8). doi:10.1371/journal.pone.0000758.

[4] Cori A, Ferguson NM, Fraser C, Cauchemez S. A new framework and software to estimate time-varying reproduction numbers during epidemics. American Journal of Epidemiology. 2013;178(9):1505–1512. doi:10.1093/aje/kwt133.

[5] Parag KV, Donnelly CA. Using information theory to optimise epidemic models for real-time prediction and estimation. PloS Computational Biology. 2020;16(7):e1007990. doi:10.1371/journal.pcbi.1007990.

[6] Parag KV, Donnelly CA. Adaptive Estimation for Epidemic Renewal and Phylogenetic Skyline Models. Systematic Biology. 2020;69(6):1163–1179. doi:10.1093/sysbio/syaa035.

[7] Dorai-Raj S. binom: Binomial Confidence Intervals For Several Parameterizations; 2014. Available from: `https://CRAN.R-project.org/package=binom`.

[8] Ferguson N, Laydon D, Nedjati Gilani G, Imai N, Ainslie K, Baguelin M, et al. Report 9: Impact of non-pharmaceutical interventions (NPIs) to reduce COVID19 mortality and healthcare demand. 2020;doi:10.25561/77482.

[9] Khalili M, Karamouzian M, Nasiri N, Javadi S, Mirzazadeh A, Sharifi H. Epidemiological characteristics of COVID-19: a systematic review and meta-analysis. Epidemiology and Infection. 2020;148:e130. doi:10.1017/S0950268820001430.

[10] Li M, Chen P, Yuan Q, Song B, Ma J. Transmission characteristics of the COVID-19 outbreak in China: a study driven by data. Epidemiology; 2020. Available from: http://medrxiv.org/lookup/doi/10.1101/2020.02.26.20028431.

[11] WHO Coronavirus Disease (COVID-19) Dashboard; 2021. https://covid19.who.int.

[12] Situation updates on COVID-19; 2021. https://www.ecdc.europa.eu/en/covid-19/situation-updates.

[13] Ward H, Atchison C, Whitaker M, Ainslie KEC, Elliott J, Okell L, et al. SARS-CoV-2 antibody prevalence in England following the first peak of the pandemic. Nature Communications. 2021;12(1):905. doi:10.1038/s41467-021-21237-w.

[14] United Nations, Department of Economic and Social Affairs, Population Division (2019). World Population Prospects 2019.; 2020. https://population.un.org/wpp.

[15] Goodrich B, Gabry J, Ali I, Brilleman S. rstanarm: Bayesian applied regression modeling via Stan.; 2020. Available from: https://mc-stan.org/rstanarm.

References

[1] Svensson Å. A note on generation times in epidemic models. Mathematical biosciences. 2007;208(1):300–311.

[2] Nouvellet P, Cori A, Garske T, Blake IM, Dorigatti I, Hinsley W, et al. A simple approach to measure transmissibility and forecast incidence. Epidemics. 2018;22:29–35. doi:10.1016/j.epidem.2017.02.012.

[3] Fraser C. Estimating individual and household reproduction numbers in an emerging epidemic. PloS One. 2007;2(8). doi:10.1371/journal.pone.0000758.

[4] Cori A, Ferguson NM, Fraser C, Cauchemez S. A new framework and software to estimate time-varying reproduction numbers during epidemics. American Journal of Epidemiology. 2013;178(9):1505–1512. doi:10.1093/aje/kwt133.

[5] Parag KV, Donnelly CA. Using information theory to optimise epidemic models for real-time prediction and estimation. PloS Computational Biology. 2020;16(7):e1007990. doi:10.1371/journal.pcbi.1007990.

[6] Parag KV, Donnelly CA. Adaptive Estimation for Epidemic Renewal and Phylogenetic Skyline Models. Systematic Biology. 2020;69(6):1163–1179. doi:10.1093/sysbio/syaa035.

[7] Dorai-Raj S. binom: Binomial Confidence Intervals For Several Parameterizations; 2014. Available from: https://CRAN.R-project.org/package=binom.

[8] Ferguson N, Laydon D, Nedjati Gilani G, Imai N, Ainslie K, Baguelin M, et al. Report 9: Impact of non-pharmaceutical interventions (NPIs) to reduce COVID19 mortality and healthcare demand. 2020;doi:10.25561/77482.

[9] Khalili M, Karamouzian M, Nasiri N, Javadi S, Mirzazadeh A, Sharifi H. Epidemiological characteristics of COVID-19: a systematic review and meta-analysis. Epidemiology and Infection. 2020;148:e130. doi:10.1017/S0950268820001430.

[10] Li M, Chen P, Yuan Q, Song B, Ma J. Transmission characteristics of the COVID-19 outbreak in China: a study driven by data. Epidemiology; 2020. Available from: http://medrxiv.org/lookup/doi/10.1101/2020.02.26.20028431.

[11] WHO Coronavirus Disease (COVID-19) Dashboard; 2021. https://covid19.who.int.

[12] Situation updates on COVID-19; 2021. https://www.ecdc.europa.eu/en/covid-19/situation-updates.

[13] Ward H, Atchison C, Whitaker M, Ainslie KEC, Elliott J, Okell L, et al. SARS-CoV-2 antibody prevalence in England following the first peak of the pandemic. Nature Communications. 2021;12(1):905. doi:10.1038/s41467-021-21237-w.

[14] United Nations, Department of Economic and Social Affairs, Population Division (2019). World Population Prospects 2019.; 2020. https://population.un.org/wpp.

[15] Goodrich B, Gabry J, Ali I, Brilleman S. rstanarm: Bayesian applied regression modeling via Stan.; 2020. Available from: https://mc-stan.org/rstanarm.
